# Fructose metabolism and its roles in metabolic diseases, inflammatory diseases, and cancer

**DOI:** 10.1186/s43556-025-00287-2

**Published:** 2025-06-23

**Authors:** Zhenhong Li, Xinzou Fan, Fan Gao, Shengguang Pan, Xiao Ma, Hao Cheng, Hiroko Nakatsukasa, Wei Zhang, Dunfang Zhang

**Affiliations:** 1 Department of Biotherapy, State Key Laboratory of Biotherapy and Cancer Center, Collaborative Innovation Center of Biotherapy, West China Hospital, Sichuan University, Sichuan Chengdu, 610041 China; 2 Center for Immunology and Hematology, Department of Biotherapy and Cancer Center and State Key Laboratory of Biotherapy, West China Hospital, Sichuan University, Sichuan Chengdu, 610041 China; 3 Laboratory of Microbiology and Immunology, Graduate School of Pharmaceutical Sciences, Chiba University, Chiba, 260-8675 Japan

**Keywords:** Fructose, Fructose metabolism, Inflammation, Tumor metabolism, Glycolysis

## Abstract

Fructose, a prevalent hexose, has become a widely used food additive, with its usage rising significantly because of socio-economic advancements and shifts in human dietary habits. Excessive fructose intake has been implicated in obesity, cardiovascular disease, metabolic syndromes, inflammation, and cancer, among other disorders. This review discusses the absorption, distribution, and metabolism of fructose and the links between fructose metabolism and major metabolic pathways. The role of fructose in metabolic diseases, including metabolic dysfunction-associated fatty liver disease, hyperinsulinemia, and hyperuricemia, is also highlighted. Furthermore, the role of fructose in the development of chronic inflammation, including gut inflammation, liver inflammation, and neuroinflammation, is discussed. Lastly, in the context of cancer development, this review summarizes the dual role of fructose in tumors, both pro- and anti-tumor effects. Future studies on the role of fructose in cancer should focus on the complexity of physiological and pathological conditions, such as the specific tumor microenvironment and metabolic status. Fructose has been shown to induce metabolic reprogramming of multiple immune cells and increase pro-inflammatory immune responses; therefore, inhibiting or promoting its metabolism may regulate immune responses. And targeting fructose metabolism may be a promising approach to treating metabolic diseases, inflammation, and cancer.

## Introduction

Humans have a natural preference for sweet foods, which induce strong sensory pleasure and can increase the chances of survival of individuals in extreme environments, leading to a stronger evolutionary advantage [1]. Fructose is the sweetest known hexose in nature, often considered a healthy sugar primarily owing to its low glycemic index and impact on blood glucose fluctuations [2, 3].

Moderate fructose consumption increases energy availability without significantly impacting insulin levels, making it a great long-term energy source, especially for physically active individuals [4]. Short-term or appropriate doses of fructose can help lower blood pressure and body mass index, improve glucose tolerance, and be a valuable nutrient [5, 6]. However, extensive research has highlighted the hazards of fructose to human health. Indeed, high fructose intake can lead to obesity, metabolic disorders, and cardiovascular and cerebrovascular diseases [3, 7–11], and has been linked to inflammation, as excessive intake induces the secretion of various pro-inflammatory cytokines [12–14]. Also fructose is inextricably associated with cancer occurrence and development [7, 15, 16].

The increasing prevalence of metabolic disorders in modern society and their association with excessive fructose intake emphasize the importance of fructose in metabolic dysregulation [17–19]. Inflammatory diseases often coincide with metabolic disorders, illustrating the systemic effects of fructose on human health [12, 20]. Additionally, the emerging link between fructose and cancer biology, both beneficial and detrimental, has made fructose a hot research topic in oncological studies [21, 22]. Therefore, herein, we reviewed the recent research on fructose metabolism and its contribution to metabolic diseases and inflammatory responses, focusing on the association between fructose intake and cancer.

First, this review outlines the absorption, transportation, and metabolic pathways of fructose, highlighting its differences from glucose metabolism. Subsequently, it analyzes the mechanism underlying the role of fructose in metabolic disorders, such as obesity and metabolic dysfunction-associated fatty liver disease (MAFLD; also known as nonalcoholic fatty liver disease, NAFLD). This review also explores how fructose triggers systemic inflammation by disrupting the intestinal barrier and inducing endoplasmic reticulum (ER) and oxidative stress. This review provides insights into the dual role of fructose in cancer in promoting tumor cell proliferation and metabolic reprogramming and potentially potentiating immune anti-tumor responses under specific conditions. A summary of the current preventative strategies for fructose-related health risks and key directions for future research are finally proposed to provide a theoretical basis for the prevention and treatment of fructose-related diseases.

## Fructose and fructose metabolism

Fructose, an isomer of glucose and the most common type of ketohexose, is found in high concentrations in honey, fruits, and vegetables. It combines with glucose in equal quantities to form sucrose, a major cyclic disaccharide in plants. In industrial food production, fructose is essential owing to its high sweetness, ease of storage, and low cost, hence, it is added to several beverages and manufactured foods as sugar in sucrose or high-fructose syrup to increase food palatability [23]. With the continuous increase in pre-made foods and drinks, the global consumption of fructose has significantly increased by an estimated 1,000% over the past 50 years [24].

The absorption and metabolism of fructose differ from those of glucose (Table 1). Glucose relies on transporter proteins such as Na^+^- and glucose-linked transporter 1 (SGLT1) for absorption active transport, whereas fructose primarily relies on glucose transporter 5 (GLUT5), which facilitates its absorption via passive diffusion [25]. The key rate-limiting enzymes involved in the metabolism of glucose and fructose also differ. This allows fructose to bypass some key rate-limiting steps and directly enter the metabolic pathways. The absorption and metabolism of glucose are strictly regulated, which makes glucose a stable source of energy, while the unregulated metabolic characteristics of fructose make it more likely to cause harm. The following sections provide an overview of fructose absorption, transport mechanisms, and metabolic pathways in different tissues.

**Table 1:** Metabolic differences between fructose and glucose

	Fructose	Glucose
Chemical Structure	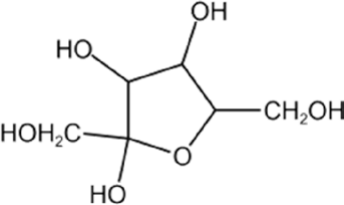	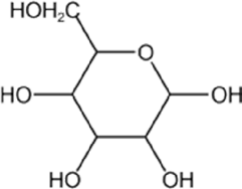
Absorption Pattern	Fructose is absorbed in the intestine via GLUT5, then transported to the liver by GLUT2 and possibly GLUT8	Glucose is absorbed in the intestine via SGLT1 and transported into the bloodstream by GLUT2
Initial Phosphorylation	Phosphorylated by Ketohexokinase to form fructose-1-phosphate	Phosphorylated by hexokinase to form glucose-6-phosphate
Key Enzyme	Ketohexokinase, Aldolase B	Hexokinase, Phosphofructokinase-1,
Rate-limiting Steps	Bypasses glucokinase and phosphofructokinase	Controlled by hexokinase and phosphofructokinase
Glycolysis	Directly catalyzed to glyceraldehyde 3-phosphate and dihydroxyacetone phosphate, thus entering glycolysis	Phosphorylated by hexokinase and enter glycolysis
TCA Cycle	Pyruvate from glycolysis enters the mitochondria, converted to acetyl-CoA, entering the TCA cycle	

*GLUT5 *glucose transporter 5, *GLUT2* glucose transporter 2, *GLUT8 *glucose transporter 8, *SGLT1* Sodium-Glucose Cotransporter 1, *TCA Cycle *Tricarboxylic Acid Cycle, *Acetyl-CoA *Acetyl Coenzyme A

### Fructose absorption and transport

Typically, fructose concentration in human peripheral blood plasma is 0.04 mM. Following oral administration of large amounts of fructose (0.5 g/kg), serum fructose levels surge 50–100-fold [26–28]. Despite this initial spike, the body reaches fasting levels within 2 h [29–31] as fructose metabolism differs from that of glucose in that the site of fructose metabolism is centralized in the intestine and liver [32, 33]. Fructose was first believed to be metabolized primarily in the liver; however, fructose enters the intestine first and is passively transported across the cell membrane from the intestinal lumen into intestinal epithelial cells via GLUT5 (also known as hexose transporter receptor, SLC2A5) localized at the brush border of intestinal epithelial cells, highlighting the intestinal role in fructose metabolism. GLUT5, a fructose-specific transporter with a greater affinity for fructose than glucose, is important for the intestinal absorption of dietary fructose. *GLUT5*-deficient mice have approximately 90% lower serum fructose levels and 75% lower levels in the jejunum than their wild-type counterparts [34]. In hepatocytes, as GLUT5 is poorly expressed in the liver, it may not be the primary transporter of fructose; rather, GLUT2 may be [35, 36]. Additionally, GLUT8 has an affinity for fructose that may contribute to fructose transport in hepatocytes [37, 38].

### Metabolism of fructose in specific organs or tissues

**Fig. 1:**
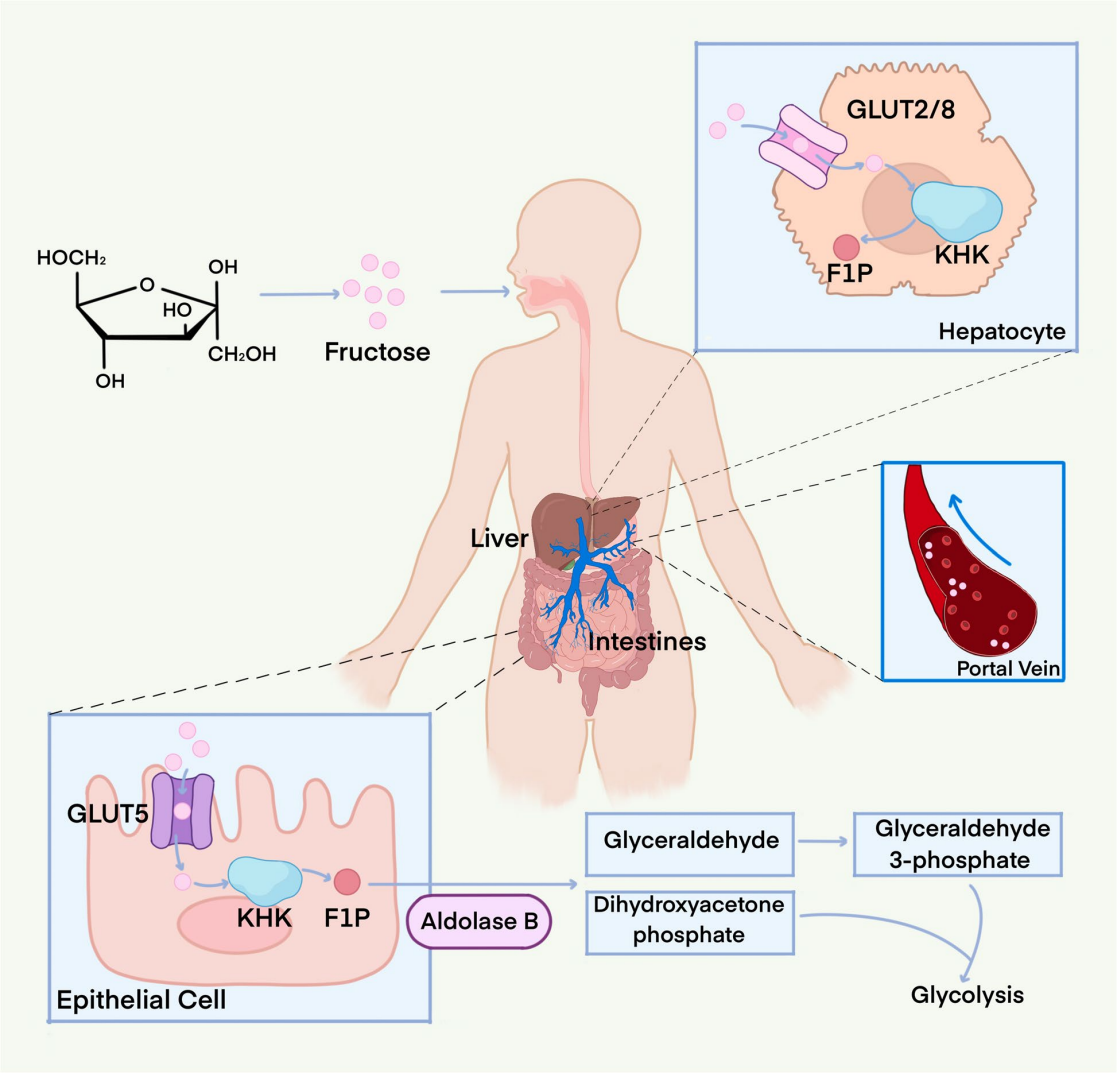
Fructose metabolism. Fructose first enters the intestine and is passively transported from the intestinal lumen across the cell membrane to the intestinal epithelial cells by glucose transporter 5 (GLUT5). Excess fructose reaches the liver through the portal vein for further metabolism. After entering hepatocytes, glucose transporter protein 2/8 (GLUT2/8) facilitates the transport of fructose, which is rapidly phosphorylated by KHK to fructose 1-phosphate and further metabolized to glucose, lactate, glycerate, and other organic acids

Fructose entering intestinal epithelial cells is phosphorylated by ketohexokinase (KHK) and converted into glucose, lactate, glycerate, and other organic acids [19]. Fructose-derived metabolites enter the liver via the portal vein [39]. If ingested at relatively low doses and rates of intake, fructose is readily cleared by the intestines [40]. However, on exceeding the intestinal absorption capacity, fructose reaches the liver, where it is metabolized [32, 41]. Reportedly, 90% of normal dietary fructose is processed primarily in the intestine [32, 42, 43]. However, if excess fructose is consumed, it will be transferred to the liver for metabolism [39, 44, 45]. Particularly, excess fructose is excreted from the intestinal epithelium via GLUT2 and enters the portal vein, reaching the liver [46]. In hepatocytes, fructose is first phosphorylated to fructose 1-phosphate by KHK, and this reaction is rapid and irreversible. The high affinity of KHK for fructose and the fact that KHK is not regulated by its end products or denaturation allows fructose to enter the liver for rapid extraction and metabolism, with negligible escape into circulation. Subsequently, fructose 1-phosphate is cleaved by aldolase B into dihydroxyacetone phosphate and glyceraldehyde (Fig. 1).

Shared similarities exist between the metabolisms of fructose in the intestine and liver; however, the ability of intestinal epithelium to metabolize fructose is very limited. Only GLUT5 transports fructose in the small intestine compared to the liver; the ability of GLUT5 to transport fructose is limited despite its increase in expression upon fructose intake [46, 47]. However, other proteins that transport fructose are expressed in the small intestine at much lower levels than GLUT5, yet are not regulated by fructose [37, 48]. Conversely, in the liver, the expression of proteins and enzymes associated with the transport and metabolism of fructose is high. In contrast, the small intestine is more inclined to rapidly translocate fructose than to metabolize it, and the expression of its associated proteins and enzymes is inducible. Considering the harmful effects of excess fructose, restricting fructose metabolism in the small intestine may be a potential protection strategy for intestinal cells [49, 50]. Therefore, the small intestine is the site of fructose transport and the initial site of metabolism, whereas the liver is the primary site of fructose metabolism. This strategy of different division of labor can be attributed to evolutionary selection.

In addition to the small intestine and liver, the remaining organs can also metabolize fructose. Indeed, previous studies have reported that the kidney can metabolize fructose [51, 52]. In healthy kidneys, the proximal tubule is the primary site of fructose metabolism, which takes up urinary fructose *via* GLUT5 and metabolizes it in the cytoplasm [53, 54]. Moreover, Na^+^- and glucose-linked transporter 5 (SGLT5), expressed only in the kidney, is also an essential transporter protein for fructose reabsorption [55–57]. Notably, the kidney produces endogenous fructose, which characterizes the metabolism of this fructose as an important risk factor for kidney injury [54, 58, 59]. Along with the liver, intestine, and kidney, which metabolize most of fructose, adipose tissues and muscles can also metabolize the remaining fructose [60–62]. Adipocytes and muscles take up fructose through GLUT5; however, they metabolize fructose primarily with hexokinase [63, 64]. As fructose is metabolized in various tissues or organs, excessive fructose intake can be harmful.

### Fructose in relationship to central carbon metabolism

Fructose is mostly phosphorylated by fructokinase to generate fructose-1-phosphate, unlike glucose, which enters glycolysis via phosphorylation by hexokinase to form glucose-6-phosphate (Table 1). Consequently, fructose-derived glyceraldehyde and dihydroxyacetone phosphate bypass glucokinase and phosphofructokinase, the key rate-limiting enzymes, and enter the glycolysis/gluconeogenic carbon pool. Glyceraldehyde is catalyzed by glyceraldehyde kinase to form 3-phosphoglyceraldehyde, which enters the glycolytic pathway. In contrast, glyceraldehyde can form dihydroxyacetone phosphate catalyzed by alcohol dehydrogenase, glycerol kinase, and glycerol phosphate dehydrogenase. Dihydroxyacetone phosphate is then converted to 3-phosphoglyceraldehyde by phosphoglycan isomerase and enters the glycolytic pathway.

The metabolic pathways are complex and overlap. Fructose is closely linked to glycolysis and impacts other metabolic pathways. For example, fructose-derived dihydroxyacetone phosphate or fructose-6-phosphate can enter the pentose phosphate pathway (PPP) through hexose phosphate isomerase [65]. Lodge et al. observed fructose labeled with C13 in PPP, specifically ribose-5-phosphate [66]. While ribose-5-phosphate and its derivatives can be used to synthesize DNA, RNA, and other important biomolecules, this shows the important role of fructose in the PPP.

Furthermore, fructose can be converted to glucose through gluconeogenesis, which occurs frequently in the kidneys [54, 67], maintaining glucose homeostasis. In contrast, glucose can be converted to fructose via the polyol pathway [68, 69]. This highlights the close relationship between fructose and glucose.

The complicated biochemical processes of fructose metabolism include intestinal absorption, liver transport, and integration into the central metabolic pathways [70]. Consuming fructose more than the body can digest induces metabolic disturbances. The liver is an essential organ for this process. Understanding these metabolic pathways and their health effects is essential, especially considering the increasing global fructose consumption.

## Role of Fructose in metabolic diseases

**Fig. 2:**
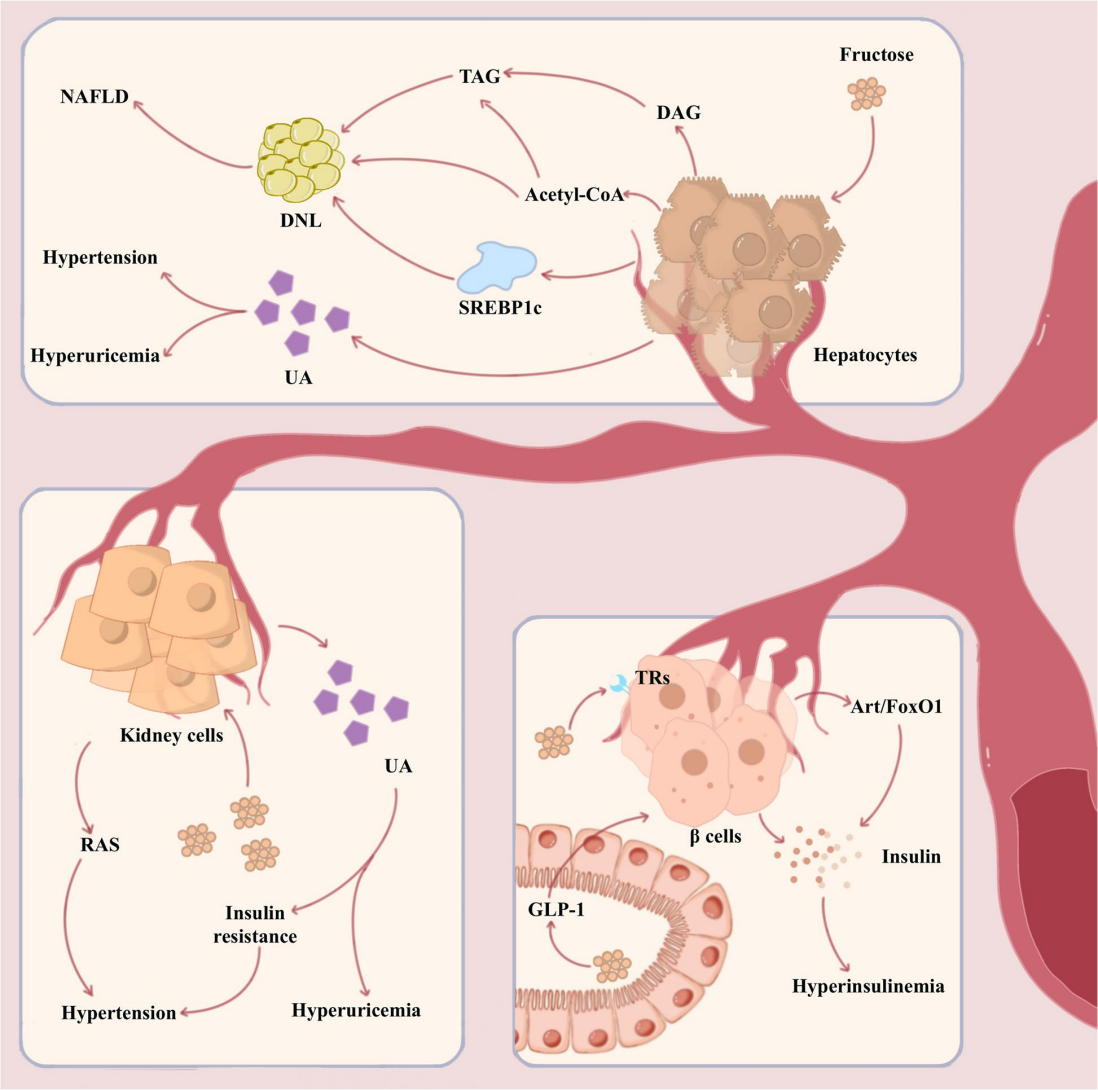
Fructose metabolism in metabolic diseases. Fructose is metabolized in the liver to form 1,2-diacylglycerol (DAG) and triacylglycerol (TAG), which ultimately contribute to de novo lipogenesis (DNL), as does acetyl-coenzyme A (Acetyl-CoA). Fructose intake also activates a key transcription factor, sterol regulatory element binding protein 1c (SREBP1c), which increases DNL levels. Dysregulation of lipid metabolism in hepatocytes because of high fructose intake ultimately causes metabolic dysfunction-associated fatty liver disease (MAFLD). High intake of fructose also causes hyperinsulinemia and reduces insulin sensitivity. Indirectly, fructose stimulates the secretion of glucagon-like peptide-1 (GLP-1) from L-cells, leading to increased insulin secretion. Fructose activates sweet taste receptors (TRs) and the protein kinase B/forkhead box protein O1 (Akt/FoxO1) pathway on β-cells, stimulating insulin secretion. In addition, fructose causes liver and kidney cells to accumulate more uric acid (UA), leading to hyperuricemia. Fructose can also contribute to hypertension by affecting the renal renin–angiotensin–aldosterone system (RAS), as well as insulin resistance caused by elevated UA

Several studies have linked consuming large amounts of fructose to the development of obesity and metabolism-related diseases, such as abnormal lipid metabolism, MAFLD, and gout [71–77]. In this section, the review summarizes several metabolic diseases associated with fructose and briefly describe their causal mechanisms (Table 2 and Fig. 2).

**Table 2:** Summary of fructose-induced metabolic diseases

Studies	Species	Methods	Results
Lanaspa et al. [9]	Sprague–Dawley rats	15% fructose fed to rats for 10 days	Fructose induces fatty liver and is dependent on xanthine oxidase
Ouyang X et al. [78]	Humans	Dietary history and paired serum and liver tissues were compared between 49 patients with MAFLD and matched with samples from 24 normal individuals	Fructose intake was nearly 2- to threefold higher in patients with MAFLD than in controls
Stanhope et al. [79]	Humans	Participants received fructose-sweetened beverages for 10 weeks at an intake of 25% of energy requirements	In overweight/obese adults, dietary fructose specifically increases de novo lipogenesis (DNL), promotes dyslipidemia, reduces insulin sensitivity, and increases visceral fat
Galderisi et al. [80]	Humans	Participants received 75 g of glucose or fructose, and plasma was collected every 10 min for 60 min	After fructose intake, insulin and GLP-1 increased more in obese adolescents than in lean adolescents
Kyriazis et al. [81]	Mice	Mice were injected intravenously with fructose (1.0 g/kg) and monitored for plasma glucose and insulin	Fructose and glucose synergistically promote insulin release
Meeprom et al. [82]	Wistar rats	Rats were fed either a normal diet or a high-fructose diet for 8 weeks; the diet in two of the treatment groups was supplemented with either 0.5% or 1.0% grape seed extract	High-fructose diet reduces insulin receptor, IRS-1, Akt, GLUT4, and adiponectin, AdipoR1, AMPK-α mRNA in skeletal muscle, causing insulin resistance
Li et al. [83]	Sprague–Dawley rats	Rats were given normal drinking water or water contain 10% fructose for 8 weeks	Fructose-fed rats exhibit obesity, fasting hyperinsulinemia, and hyperleptinemia, but no fasting or postprandial hyperglycemia
Cabral et al. [84]	Sprague–Dawley Rats	Both groups of rats were fed 20% fructose in the drinking water for 14 days. One group was started on a high-salt diet (8% NaCl) after 7 days of fructose treatment	20% fructose in the diet and high salt synergistically contribute to higher blood pressure
Johnson et al. [85]	Humans	Researchers analyzed a previously published randomized controlled study that included 33 healthy male adults who ingested 200 g of fructose daily for 2 weeks	Fructose intake leads to increased serum UA levels, decreased serum ionized calcium, mildly increased PTH, decreased urinary pH, increased urinary oxalate and decreased urinary magnesium

*MAFLD *Metabolic dysfunction-associated fatty liver disease, *GLP-1 *glucagon-like peptide-1, *IRS-1 *insulin receptor substrate-1, *Akt *protein kinase B, *GLUT4 *glucose transporter 4, *AdipoR1 *adiponectin receptor R1, *AMPK *AMP-activated protein kinase, *UA *uric acid, *PTH *parathyroid hormone

### Fructose consumption promotes obesity

The prevalence of obesity has been steadily increasing, with the same trend seen for the consumption of fructose, potentially linking fructose and obesity [2]. In a study, participants who consumed soda with high-fructose corn syrup for three weeks experienced notable weight gain, with similar outcomes observed with sucrose [86, 87]. These early studies used only mixtures containing fructose and ignored whether fructose alone had a corresponding effect. In later experiments, researchers realized that fructose did not appear to have a direct effect on weight [88–90]. In a recent test of sugary beverages involving 131 participants, those who consumed glucose and high-fructose corn syrup gained significantly more weight, whereas no significant difference was observed in weight among those who consumed only fructose [91]. These findings indicate that fructose did not cause weight gain under equal caloric intake [92] and may indirectly increase body weight by increasing energy intake [93]. In animal studies, long-term fructose intake induced leptin resistance, which, in turn, promoted energy intake and led to obesity [71, 94]. It has also been claimed that fructose intake reduces leptin concentrations but does not have a significant effect on weight [91]. In addition, fructose may also promote the survival of intestinal cells and increase the length of intestinal villi, which in part enhances nutrient absorption and contributes to obesity [95]. Interestingly, fructose mediates a survival switch in organisms that aids in storing energy when resources are lacking (similar to the state of animals preparing for hibernation) [93]. But in the present resource-rich world, this protective mechanism has harmed organisms. Thus, the obesity caused by fructose may not be a direct effect, but rather an indirect promotion of the energy intake of the body through other pathways.

### Fructose impacts lipid metabolism and MAFLD

MAFLD, a chronic liver disease closely related to metabolic disorders, has become a major public health challenge, the risk of which can be significantly increased by high-fructose intake [78, 96, 97].

Enhanced de novo lipogenesis (DNL) is a major cause of MAFLD [98]. Fructose promotes DNL through several mechanisms. Fructose is metabolized in the liver to the DNL substrates dihydroxyacetone, phosphate, and glyceraldehyde. In the presence of α-phosphoglycerol dehydrogenase, dihydroxyacetone phosphate produces glycerol phosphate. The reduced glycerophosphate produces 1,2-diacylglycerol (DAG) through various enzymatic reactions.

In a process catalyzed by acyltransferases, DAG interacts with acyl-coenzyme A to form triacylglycerol (TAG), whose levels are associated with DNL [99, 100]. In contrast, TAG is involved in steatosis via binding to lipid droplets or in the formation of very low-density lipoprotein conjugates secreted from the liver into circulation. Glyceraldehyde is a fructose derivative phosphorylated to 3-phosphoglyceraldehyde by triphosphate kinases and enters the glycolytic process [19]. Following glycolysis, 3-phosphoglyceraldehyde is metabolized to pyruvate, which is then oxidized and decarboxylated by the pyruvate dehydrogenase complex to form acetyl-coenzyme A (acetyl-CoA). Under energy-deficient conditions, acetyl-CoA enters the tricarboxylic acid cycle (TCA cycle) and is metabolized to release energy. Under energetic conditions, acetyl-CoA acts as a substrate and participates in DNL. Acetyl-CoA can also be carboxylated to form malonyl-CoA, which inhibits the transfer of fatty acids from carnitine palmitoyl transferase (CPT1A) to mitochondria for oxidation, increasing the fatty acid stocks available for TAG production [101].

In addition, fructose intake increases DNL levels by activating key transcription factors [102]. Sterol regulatory element-binding protein 1c (SREBP1c) is a regulator of adipose synthase, whose activity increases upon fructose intake. Dihydroxyacetone phosphate is a fructose metabolite that activates the mTORC1 pathway and promotes SREBP1c activation [103]. However, SREBP1c is regulated at the transcriptional and post-translational levels by nutrients and hormones [19, 45]. In summary, fructose promotes DNL and is an important factor in MAFLD [17, 104, 105]. A high-fructose diet increases the rate of fasting DNL from 2 to 9% [106]. In abdominally obese men on a habitual diet for over 12 weeks, 75 g/day fructose intake significantly increased DNL in the fasting state and 4 to 8 h following a meal [107, 108]. Similarly, nine days of isocaloric fructose restriction as part of a normal diet resulted in a significant decrease in DNL in 37 of 40 children with obesity [109].

### Fructose contributes to hyperinsulinemia and insulin resistance

Insulin regulates blood glucose levels, which are secreted by pancreatic beta cells [110]. When insulin is overproduced or not removed promptly, it may manifest as hyperinsulinemia [111–115]. Insulin resistance is defined as the inability of a given amount of insulin to promote normal glucose uptake and utilization and can also be understood as reduced sensitivity and responsiveness to insulin action [116, 117]. Studies have shown a strong link between hyperinsulinemia and insulin resistance, a precursor to the development of diabetes mellitus [118–120].

Insulin resistance co-occurs with hyperinsulinemia because of defective insulin action [119, 121]. Fructose intake can trigger hyperinsulinemia and insulin resistance [80, 122–124]. Indeed, high fructose intake reduces insulin sensitivity and glucose tolerance in rats [125, 126]. Fructose also directly activates sweet taste receptors on beta cells, promoting glucose-stimulated insulin secretion in humans and mice [81]. The authors also found that this mechanism only occurs in the presence of glucose, suggesting a synergistic interaction between fructose and glucose [81, 127]. However, fructose cannot directly promote insulin secretion because of the absence of GLUT5 in beta cells, and fructose intake stimulates glucagon-like peptide-1 (GLP-1) secretion via GLUT5-containing L-cells in the gut, thereby increasing insulin secretion, an effect more pronounced in individuals with obesity [80, 128, 129]. Furthermore, Li et al. found that fructose activates the protein kinase B/forkhead box protein O1 (Akt/FoxO1) pathway in beta cells, which mediates the action of leptin on beta cells and promotes insulin secretion [83, 130–132]. High fructose intake can reduce the expression of insulin receptors, insulin receptor substrate-1, protein kinase B (Akt), and glucose transporter 4 (GLUT4), directly inducing insulin resistance [133, 134]. Fructose intake also decreases mRNA expression of adiponectin, adiponectin receptor R1 (AdipoR1), and AMP-activated protein kinase (AMPK)-α [134, 135] and reduces adiponectin, which correlates with insulin sensitivity [82, 136, 137]. Evidently, fructose intake has persistent adverse effects in individuals with hyperinsulinemia [123].

### Fructose causes hyperuricemia and hypertension

Uric acid (UA) is the end product of purine metabolism, not further degraded as the body lacks the enzyme uric acid oxidase [138–141]. Under normal conditions, UA acts as an antioxidant that provides several benefits [142–144]. However, an abnormal increase in UA can lead to hyperuricemia, negatively affecting human health [145–148]. Examples include fat accumulation and steatosis [149, 150]. Fructose intake is associated with elevated fasting UA levels [85, 151–153] because of the unique metabolic processes of fructose. After ingestion, fructose is rapidly extracted from the liver and phosphorylated to fructose 1-phosphate by KHK. This reaction is rapid, not controlled by negative feedback regulation, and consumes a large amount of adenosine triphosphate (ATP) in a short period [154]. ATP exhaustion is accompanied by a large production of adenosine monophosphate (AMP) [155, 156]. The large amount of AMP produced is catalyzed by adenosine deaminase, yielding hypoxanthine. Hypoxanthine is eventually hydrolyzed to UA by two oxidations of xanthine oxidase, which increases UA levels in the body. Fructose intake reduces water loss by stimulating pressin secretion, reducing urine volume [157]. Moreover, large amounts of UA produced by fructose metabolism are not readily excreted through the urine, elevating UA levels. Furthermore, fructose-induced insulin resistance increases UA levels by decreasing UA excretion and upregulating inflammation [158, 159].

Hypertension is an extremely complex disease with unclear predisposing factors [84, 160–162]. Although hypertension is a multifactorial disease, fructose intake may be an important factor in regulating hypertension [3, 163–166]. Previous findings indicated that fructose intake increased blood pressure compared to glucose intake, along with oxygen consumption and respiratory quotient [79, 167, 168]. One study showed that four weeks of continuous fructose feeding increased the mean arterial blood pressure in rats [169]. Similarly, another study reported that rats developed hypertension after three weeks of a fructose diet [170]. Furthermore, the offspring of rats exposed to a 60% high-fructose diet during pregnancy and lactation showed an increased risk of hypertension [171]. How does fructose increase blood pressure? Interestingly, fructose has been shown to regulate blood pressure through UA [3, 163, 172, 173]. Fructose intake can increase UA levels, which may cause endothelial dysfunction by contributing to insulin resistance, which increases the risk of hypertension [174, 175]. UA can also stimulate the renal renin–angiotensin–aldosterone system, inducing the proliferation of vascular smooth cells and endothelial dysfunction [176]. For every 1 mg/dL increase in serum UA levels, the prevalence of hypertension increases by 13% [177]. Pharmacological intervention studies have shown that febuxostat, a xanthine oxidase inhibitor, prevents fructose-induced hypertension by reducing the UA levels [178, 179]. Furthermore, fructose increases blood pressure through several other pathways, such as sodium handling, activation of the renal sympathetic nervous system, and synergy with salt [72, 180–182].

## Role of fructose in systemic chronic inflammation

**Fig. 3:**
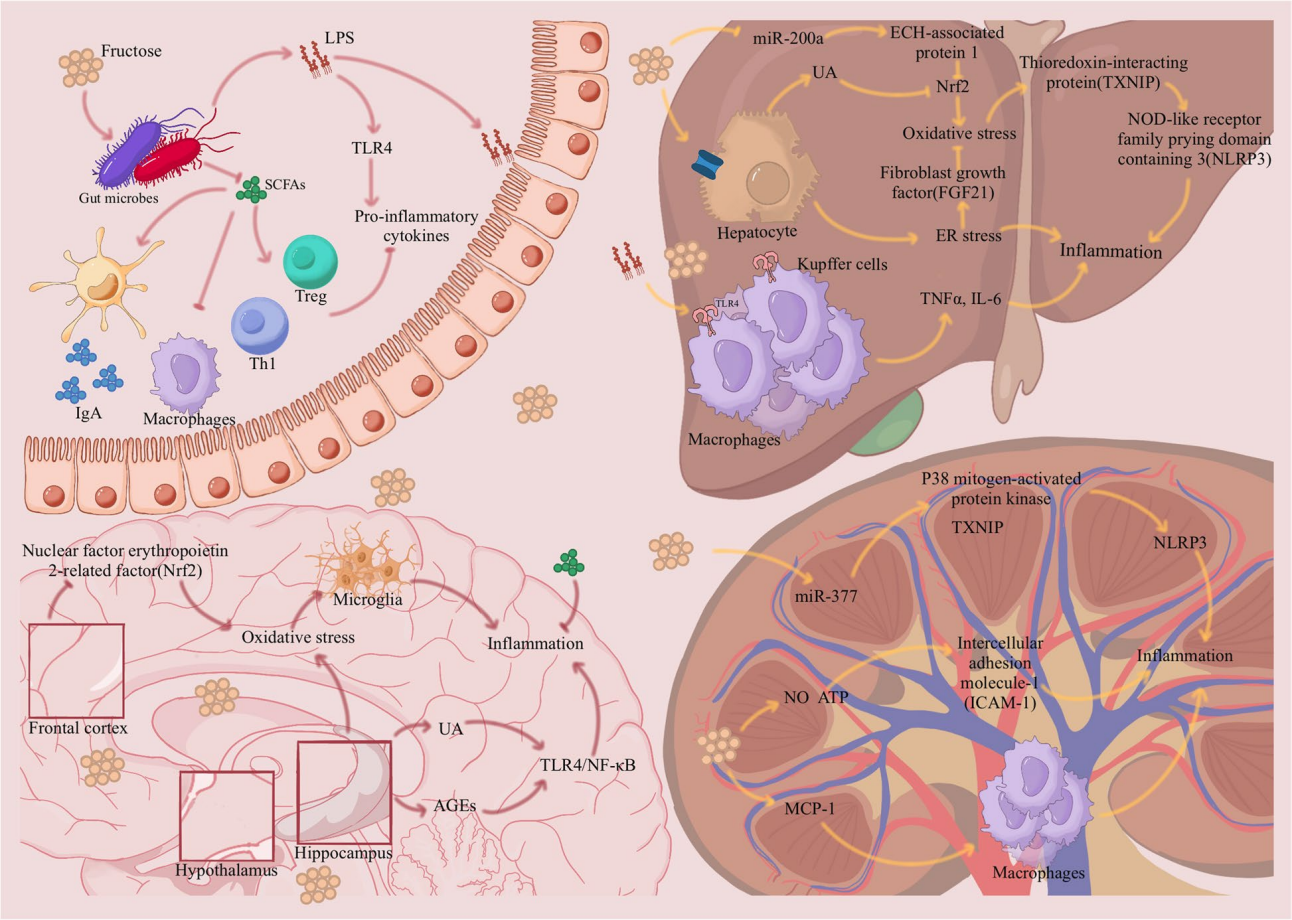
Fructose promotes systemic chronic inflammation. High fructose intake leads to a leaky gut, which in turn induces an inflammatory response. Abnormal gut microbiology reduces the secretion of short-chain fatty acids (SCFA) and promotes the release of lipopolysaccharides (LPS), which further activates the Toll-like receptor 4 (TLR4), thereby mediating various inflammatory effects. In the liver, fructose induces oxidative stress and endoplasmic reticulum (ER) stress in hepatocytes, ultimately triggering inflammation, in a variety of ways, including by promoting uric acid (UA) accumulation, while fructose also activates secretion of inflammatory factors by hepatic macrophages. In addition, fructose intake results in elevated levels of UA and advanced glycation end products (AGEs) in mouse hippocampi, which in turn induces hippocampal inflammation via the TLR4/NF-κB pathway. Fructose also activates resident microglia and secretes inflammatory factors by causing oxidative stress in the brain. At the same time, SCFA relieves the inflammatory response in the brain. Fructose in the kidney ultimately mediates inflammation through elevated microRNA-377 (miR-377) and reduced nitric oxide (NO) and ATP levels while recruiting monocytes or macrophages by inducing monocyte chemotactic protein 1(MCP-1)

Increased fructose intake is associated with several inflammatory diseases [183–185]. Fructose is metabolized and absorbed in various body parts, triggering an inflammatory response, including gut inflammation, liver inflammation, and neuroinflammation (Table 3 and Fig. 3) [186–189].

**Table 3:** Overview of fructose-induced inflammatory responses

Studies	Species	Methods	Results
Wang et al. [20]	Sprague–Dawley rats	Rats were administered pure fructose at a dose of 0, 2.6, 5.3, and 10.5 g/kg/day for 20 weeks	High intake of fructose increased UA, pro-inflammatory cytokines, intestinal permeability, and lipid accumulation in the liver and induced an inflammatory response in the pancreas and colon
Lodge et al. [66]	C57BL/6 J mice	Mice were supplemented with 30% fructose, glucose or no supplementation in their drinking water for 24 or 32 weeks	Chronic fructose exposure caused liver injury and increased anti-inflammatory and resolution associated genes in KC
Tan et al. [190]	Balb/c mice	Mice were fed either a high-fructose diet (60%), a high-fat (60 kcal%), or a normal diet for 8 weeks	Abnormalities in the intestinal structure were found in both the high-fructose and high-fat groups, and infiltration of inflammatory cells was observed
Seki et al. [191]	Fisher 344 rats	Rats were fed either CSAA diet + water, CDAA diet + water, and CDAA + water containing 20% fructose for 10 weeks	Fructose exacerbates liver fibrosis through increased intestinal permeability and contributes to the progression of MASH
Li et al. [192]	C57BL/6N mice	Mice were fed a standard diet (3.4 kcal/g) or a high-fructose diet (35% fructose-derived calories) for 4 or 8 weeks	High-fructose diet caused the hippocampal neuroinflammatory response, reactive gliosis, and neuronal loss in rats
Xu et al. [193]	ICR mice	Fructose-fed mice were given drinking water with 30% fructose solution and control mice were given normal drinking water for a total of 56 days	Fructose feeding induced hippocampal microglia activation with neuroinflammation through the activation of the TLR4/NF-κB signaling
Chen et al. [194]	ICR mice	Mice were given ordinary drinking water or 10% fructose solution for 8 weeks	The rats fed fructose showed a significant decrease in epithelial cell brush border and renal tubules, with tubulointerstitial infiltration primarily involving mononuclear cells and macrophages
Kovačević et al. [195]	Wistar rats	For 9 weeks, rats in the control group were fed standard food and drinking water, and rats in the fructose group were fed the same food and a 10% fructose solution	Rats fed fructose showed enhanced VAT mass, elevated nuclear accumulation of NF-κB, and elevated IL-1β expression, but not TNFα expression

*KC *Kupffer cells, *CSAA *choline-supplemented/L-amino acid, *CDAA *choline-deficient/L-amino acid, *MASH *Metabolic dysfunction-associated steatohepatitis, *TLR4 *Toll-like receptor 4, *VAT *visceral adipose tissue, *NF-κB *nuclear transcription factor κB

### Excessive fructose intake leads to intestinal damage and inflammation

The gut, the body’s largest barrier to the external environment, is important in protecting the organism from harm and the primary site of fructose absorption [196]. Excess fructose intake can cause intestinal barrier damage and endotoxemia [197–200]. Damage to the intestinal barrier increases the exposure to various metabolites, triggering an inflammatory response [201]. Excess fructose intake has also been shown to cause nitration of intestinal tight junction and adherent junction proteins, which can lead to an increased leaky gut [202, 203]. A large influx of antigens and other macromolecules into the barrier causes local or systemic inflammation [204, 205].

In addition, dysregulation of the gut microbiota promotes gut barrier damage and inflammatory responses [206]. The gut microbiota is the “second genome” and plays a significant role in the body’s metabolism and immunity [190]. Tan et al. reported elevated levels of *Bacteroides*, *Akkermansia*, *Lactobacillus*, and *Ruminococcus* in the intestines of rats after fructose feeding, which may be associated with inflammation [190]. Similar studies have shown that a fructose diet increases the abundance of *Bacteroides*, *Bifidobacterium*, and *Marvinbryantia* [20, 198]. In contrast, one study showed that mice fed a high-fructose diet had a lower proportion of *Bacteroidetes* and an increased proportion of *Proteobacteria* [88]. Despite the differences between the results of previous studies, alterations in the gut microbiota do influence intestinal inflammation and damage to the intestinal barrier.

Metabolites of the gut microbiota, such as lipopolysaccharides (LPS) and short-chain fatty acids (SCFAs), are important signaling mediators [207]. Dysbiosis of the gut microbiota promotes the release of LPS (Parabacteroides is the main source of LPS), which in turn activates the Toll-like receptor 4 (TLR4) and exacerbates intestinal inflammation by inducing the release of pro-inflammatory cytokines [208–210]. At the same time, because of the altered intestinal permeability, LPS circulates through the portal vein to the liver and induces secretion of the inflammatory factor tumor necrosis factor-alpha (TNF-α) by activating TLR4 on macrophages [40, 211, 212]. SCFAs are important signaling molecules for metabolic and immune regulation [207, 213, 214]. Acetate, an SCFA, induces retinoic acid production in dendritic cells, which further promotes the intestinal IgA response and protects the gut from inflammatory damage [215–217]. Similarly, n-butyrate, another SCFA, induces intestinal macrophages to reduce the secretion of pro-inflammatory mediators such as nitric oxide (NO), interleukin-6 (IL-6), and IL-12 by inhibiting histone deacetylases [218, 219].

SCFAs also affect other immune cells in the gut. For example, Sun et al. demonstrated that SCFAs activate Th1 cell STAT3 and mTOR and upregulate transcription factor B lymphocyte-induced maturation protein 1 (Blimp-1), which can induce the production of IL-10 [220]. Notably, SCFAs can induce the differentiation of intestinal regulatory T cells, which maintain intestinal homeostasis [221–224]. Overall, SCFAs have a positive effect on gut homeostasis and immunity; however, fructose intake appears to reduce SCFAs in the gut [192]. And indirectly, fructose intake affects gut stability and induces inflammation.

### Excessive fructose intake induces inflammation, resulting in liver injury

Excessive fructose intake leads to MAFLD and contributes to metabolic dysfunction-associated steatohepatitis (MASH), which may progress to liver fibrosis, cirrhosis, or even liver cancer [191, 225]. Recently, several studies have revealed fructose-induced inflammation in the liver [66, 226–229]; however, the induction mechanism is complex. Hepatocytes are particularly vulnerable to ER stress. A chronic fructose diet affects lipid metabolism and the production of very low-density lipoproteins, which leads to ER stress and the unfolded protein response (UPR). ER stress induces inflammation, oxidative stress, and apoptosis [230–232]. Oxidative stress induced by fructose can induce inflammation via the accumulation of oxygen reactive species (ROS) as it can activate some inflammatory pathways, including nuclear factor kappa B (NF-κB) and C-Jun amino terminal kinase (JNK) [233, 234]. A high-fructose diet can inhibit the ER stress-induced production of fibroblast growth factor 21 (FGF21), reducing oxidative stress [235]. In addition to cellular stress, UA is an important inflammatory trigger [236]. UA in the liver contributes to oxidative stress and inflammation by inhibiting nuclear factor erythroid 2-related factor (Nrf2) and the production of thioredoxin, leading to the activation of the NOD-like receptor family pyrin domain containing 3 (NLRP3) inflammasome [237, 238]. Furthermore, a more in-depth study showed that fructose intake reduced microRNA-200a (miR-200a), targeting Kelch-like ECH-associated protein 1 (Keap1) and inhibiting the Nrf2 antioxidant pathway, thereby triggering the thioredoxin-interacting protein (TXNIP)-activated NLRP3 inflammasome, ultimately inducing liver inflammation [239]. The liver also contains various macrophages, including Kupffer cells (KC) and other recruited monocytes or macrophages, which typically exhibit a pro-inflammatory phenotype [240–242]. It has also been reported that a fructose diet can activate TLR4 on KC, which elevates ROS, induces inflammation, and induces hepatocyte necrosis by increasing the expression of TNF-α and IL-6 [243–245]. This process may involve the fructose-induced increase in fatty acids, such as acylcarnitine and palmitate [245–248].

### Fructose triggers neuroinflammatory responses in key brain regions

Fructose-induced inflammation has been widely reported in various tissues, including nervous tissue. Fructose-induced neuroinflammation has attracted attention [193, 249–251]. Cells in the brain can directly metabolize fructose; GLUT5 expression has been detected in the hippocampal microglia [252, 253]. The hippocampus, the memory center of the brain, is vital for learning and memory. Recent studies have found that excessive fructose intake damages hippocampal function [254, 255]. Indeed, excessive fructose intake blunts hippocampal plasticity and reduces hippocampal weight, which reflects functional changes in brain cells [256–260]. Fructose intake causes an increase in CLUT5 in the hippocampus of mice and an increase in UA levels; UA can induce hippocampal inflammation via the TLR4/NF-κB pathway [261, 262]. Additionally, accumulation of toxic compounds and advanced glycation end products (AGEs) because of fructose intake has been linked to inflammation [12, 263–265]. Mastrocola et al. reported that in mice fed a 60% fructose diet for 12 weeks, carboxymethyl lysine, an AGE that accumulates in hippocampal neurons, is induced and activates NF-κB signaling [266]. Fructose-induced hippocampal inflammation is associated with oxidative stress in the brain, which activates the resident microglia and secretes inflammatory factors [267–270]. Cigliano et al. found an increase in lipid peroxidation and nitro-tyrosine in the hippocampus of rats after 2 weeks of fructose feeding, suggesting the presence of oxidative stress damage. The author also detected an increase in TNF-α levels, with a positive correlation with oxidative stress [270]. Indirect mechanisms have also been reported in the hippocampus. Li et al. found that high fructose-induced intestinal dysregulation induces hippocampal neuroinflammation in mice, which can be alleviated by SCFA supplementation [192]. What is surprising is that excessive maternal fructose intake can damage the hippocampus of the offspring, an effect linked to reduced expression of the brain-derived neurotrophic factor (*BDNF*) gene [271].

In addition to the hippocampus, fructose-induced inflammation has also been observed in other brain parts. The hypothalamus, a component of the mesencephalon, is the center for regulating visceral and endocrine activity, where astrocytes play an important role. Inflammatory responses in this region cause various metabolic disorders [272–275].

Li et al. revealed that fructose intake induced hypothalamic astrocytosis and inflammation by activating the TLR4/NF-κB pathway, resulting in neurological damage in the hypothalamus [276]. Similarly, fructose-induced inflammatory responses have been observed in the frontal cortex. As the frontal cortex is the latest area of the brain to mature, its development is susceptible to dietary influences [277–280]. Indeed, a fructose diet for two weeks has been shown to negatively affect the nuclear factor (erythroid derived 2)-like 2 (Nrf2) pathway in the frontal cortex of rats, impairing the brain’s antioxidant defense system and causing oxidative stress and synaptic dysfunction in the frontal cortex [281].

### Inflammatory effects of fructose in other body parts

Fructose induces inflammation in various tissues and organs [206]. The kidney is primarily responsible for filtering impurities and metabolic waste from blood. Various studies have demonstrated a strong correlation among fructose intake, kidney damage, and inflammation [178, 194, 282–284]. Wang et al. showed that a fructose diet increased microRNA-377 (miR-377) expression in the kidney and miR-377-induced p38 mitogen-activated protein kinase phosphorylation and TXNIP expression, which in turn activated the NLRP3 inflammasome, ultimately leading to inflammation [285]. Another study showed that fructose induced the synthesis of monocyte chemotactic protein 1 (MCP-1), recruitment of monocytes or macrophages, and oxidative stress in proximal tubular cells; this effect depends on KHK [178, 286]. Furthermore, fructose-induced decreases in renal endothelial NO and ATP levels upregulates the inflammatory molecule intercellular adhesion molecule-1 (ICAM-1) expression [287].

Similarly, a comparable mechanism has been reported in adipocytes. Excessive fructose intake induces the expression of MCP-1 and ICAM-1 in adipocytes, leading to an increase in macrophage infiltration, further contributing to inflammation [287–289]. Furthermore, fructose increases leptin levels, inducing inflammation in adipocytes by releasing ROS [94, 290, 291]. Additionally, a fructose diet increases visceral adipose tissue mass, NF-κB accumulation, and elevated IL-β in rats [195]. A fructose-rich diet can also affect pancreatic islet cells, leading to hyperinsulinemia and insulin resistance. Moreover, fructose induces an inflammatory response in the pancreatic islet cells, and its intake increases the size and number of pancreatic islets; fructose-induced UA stimulates inflammatory mediators and oxidative stress in pancreatic islet cells [89, 292, 293].

## Co-mechanisms of fructose in metabolic diseases or inflammation

Previous sections have detailed fructose's role in various metabolic diseases; the corresponding studies are summarized in Table 2. Although a direct causal relationship between fructose and obesity remains to be confirmed, it has been shown to play an important role in the development of lipid metabolic disorders, MAFLD, and metabolic syndromes (including hyperinsulinemia, insulin resistance, hyperuricemia, and hypertension) (Fig. 2) [17, 91, 294, 295].

Hyperuricemia is a key contributor to various metabolic diseases. Fructose metabolism induces the production of uric acid, which directly causes gout, exacerbates insulin resistance, and participates as an inflammatory factor in the occurrence of MAFLD and hypertension [149, 179, 236].

Inflammation is an important mechanism via which fructose exposure leads to metabolic diseases. Excessive fructose can induce inflammatory responses in organs, such as the gut, liver, brain, and kidneys (Table 3). Activation of the TLR4/NF-κB pathway is a common mechanism of inflammatory responses. In the gut, fructose promotes intestinal flora dysregulation, increases LPS production, activates the TLR4/NF-κB pathway, and triggers intestinal inflammation [208, 210]. In the liver, factors such as disordered fructose metabolism, elevated uric acid, and fatty acid accumulation can activate TLR4 in KC, which in turn activates the NF-κB pathway and releases pro-inflammatory cytokines [244]. Similarly, in brain regions such as the hippocampus, the fructose metabolite UA can induce neuroinflammation through the TLR4/NF-κB pathway [261].

In conclusion, fructose plays a complex role in various metabolic diseases, with inflammation being an important contributing pathogenic mechanism. A deeper understanding of fructose metabolism and the inflammatory responses it triggers could help develop more effective strategies to prevent and treat related metabolic diseases. Further studies remain warranted to elucidate the mechanisms underlying fructose action in different organs and tissues and develop targeted interventions.

## Complex relationship between fructose and cancer

The link between fructose and tumor has also attracted extensive attention in recent years. Long-term high fructose consumption is implicated in a range of cancers, and its role varies depending on the type of cancers [296–298]. This section collects epidemiological evidence on the relationship between fructose and tumors and discusses the role of fructose in tumor development.

### Epidemiological studies on high-fructose diets and cancer risk

Excessive fructose intake is linked to various metabolic disorders, as confirmed by epidemiological studies [299–301]. The relationship between fructose and cancer has been a research focus. A previous investigation included approximately 1.2 million participants and over 3000 pancreatic cancer cases to investigate the relationship between fructose, carbohydrates, glycemic index, and pancreatic cancer risk [302]. The authors revealed for the first time the links between fructose intake and pancreatic cancer risk [302]. Another study investigated the relationship between fructose from diet and colorectal cancer and reported a higher incidence of colorectal cancer among those consuming fructose at higher levels [303]. Likewise, a 12-year epidemiological study involving a cohort of Canadian women reported that a high intake of sugar-sweetened beverages was associated with a significantly elevated risk of endometrial and ovarian cancer [304]. Although the authors did not specifically identify fructose, most sugary drinks contain fructose as an ingredient. Fructose also appears to be associated with poor patient prognosis. For example, a study analyzed the relationship between the intake of different types of carbohydrates and breast cancer-specific mortality in patients diagnosed with breast cancer [305]. The data from 8,932 breast cancer patients followed for more than a decade showed a highly significant positive association between higher fructose intake and the risk of breast cancer-specific mortality [305].

Nevertheless, not all studies have reported a positive association between fructose and cancer risk. For instance, in a survey of 3,184 adults aged 26–84 years, no significant correlation was found between fructose intake and the incidence of obesity-related cancers, nor an association with the risk of any site-specific cancer [306]. Following the same trend, a meta-analysis pooled multiple prospective cohort studies and found that while excessive total sugar and fructose intake were associated with all-cause and cardiovascular disease mortality, no association was found with cancer mortality [307].

These conflicting results highlight the degree of complexity in the fructose-cancer relationship. Fructose significantly influences people's diets, and its effects may vary based on age, geography, or dietary habits. [303, 304, 306]. Conversely, fructose may involve multiple mechanisms in its effects on cancer in the body.

### Role of fructose in tumors

**Fig. 4:**
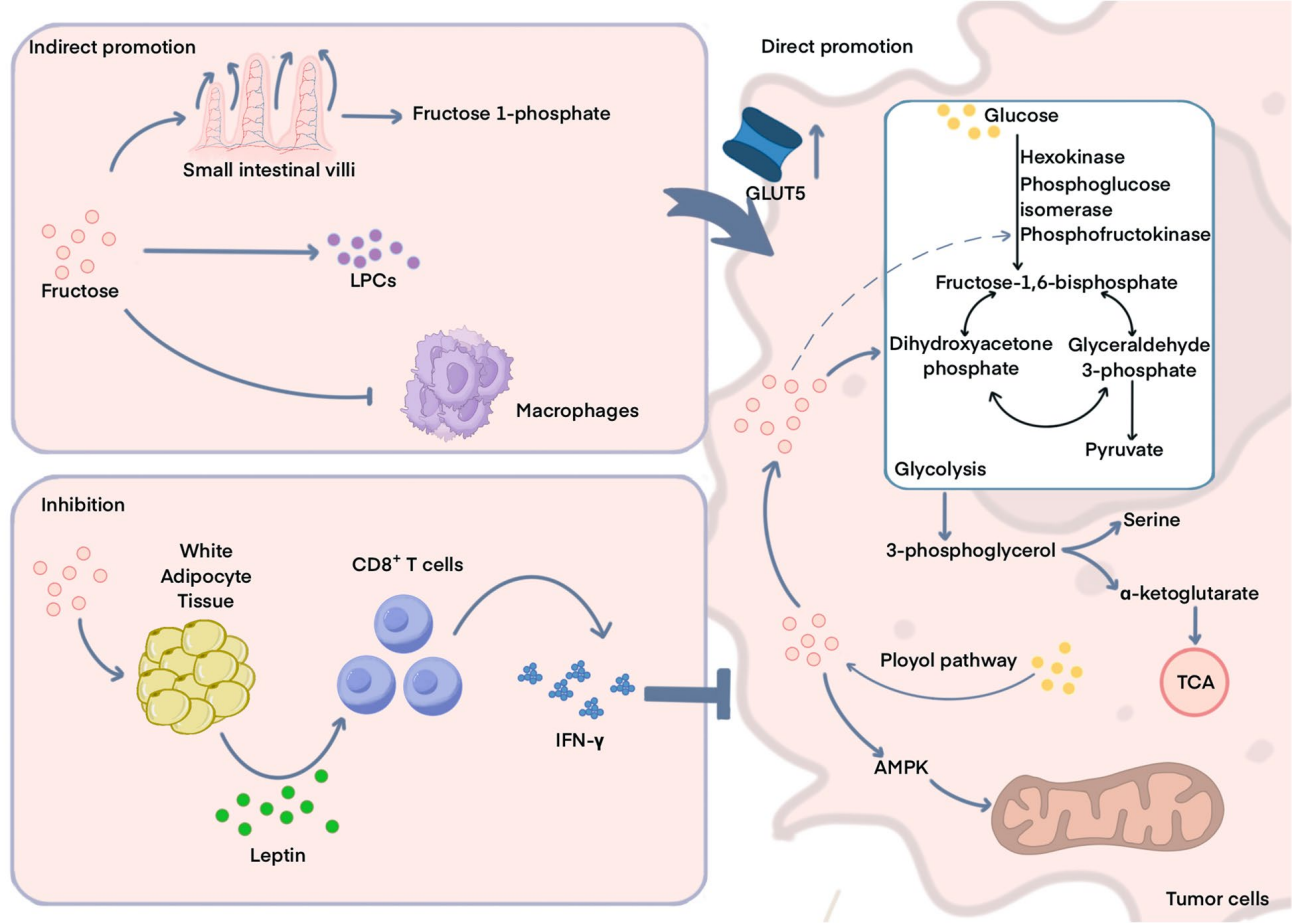
The dual role of fructose in cancer. The relationship between fructose and cancer is complex. Fructose promotes the growth of small intestinal villi, enhancing their absorption capacity. Fructose is rapidly converted to fructose 1-phosphate by ketohexokinase (KHK), and upregulates the expression of glucose transporter 5 (GLUT5). In addition, the metabolism of fructose through the liver leads to a large increase in circulating nutrients such as lysophosphatidylcholines (LPCs). These processes indirectly provide appropriate substrates for central carbon metabolism during tumor proliferation, and fructose also inhibits the polarization of tumor-associated macrophages, further promoting tumor growth. Because of the special metabolic process of fructose, fructose enhances tumor metabolism through glycolysis, the serine synthesis pathway, polyol pathway and so on. fructose also enhances the function of mitochondria through the AMP-activated protein kinase (AMPK) after absorbed by tumor cells, and enhances fatty acid synthesis by up-regulating the expression of glucose transporter 5 (GLUT5). These processes will directly promote tumor growth. On the contrary, fructose can induce adipocytes to secrete leptin, which can act on CD8^+^ T cells and enhance the anti-tumor activity of CD8^+^ T cells

Fructose plays complex and diverse roles in tumors (Table 4 and Fig. 4). Tumor metabolism favors aerobic glycolysis, a process through which ample energy is supplied to the tumor to support the rapid proliferation of tumor cells.

**Table 4:** Complex role of fructose in tumor development

Studies	Role in tumors	Specific Mechanism
Jeong S et al. [16]	Directly Promotion	High fructose enhances SSP, increasing α-ketoglutarate production, which supports leukemia cell proliferation
Goncalves MD et al. [21]	Directly Promotion	Fructose promotes glycolysis and DNL, increasing fatty acid synthesis for tumor cell growth
Bu P et al. [22]	Directly Promotion	Upregulated aldolase B enhances fructose metabolism, promoting liver metastasis of colorectal cancer
Taylor S R et al. [95]	Indirectly Promotion	Fructose improves intestinal cell survival and increases the length of intestinal villi. The increase in the length of intestinal villi enlarged the surface area of the mouse intestines, which increased the rate of nutrient absorption and thus promoted tumor growth
Kuehm LM et al. [313]	Indirectly Promotion	Melanoma tumors in mice on the high-fructose diet were resistant to immunotherapy and showed increased expression of the cytoprotective enzyme HO-1
Fowle-Grider R [314]	Indirectly Promotion	fructose supplementation increases circulating nutrients such as LPCs, which can enhance tumor growth through a cell non-autonomous mechanism
Yan H et al. [315]	Indirectly Promotion	Fructose inhibits M1 macrophage polarization, reducing anti-tumor activity by altering calcium ion signaling
Chen WL et al. [316]	Directly Promotion	AML cells are prone to fructose utilization with an upregulated fructose transporter GLUT5, which compensates for glucose deficiency
Fang J H et al. [298]	Directly Promotion	Fructose activates AMPK, enhancing tumor angiogenesis and growth in liver cancer
Kang Y L et al. [317]	Directly Promotion	Polyol pathway increases endogenous fructose, activating KHK-A and inducing epithelial-mesenchymal transition, promoting cancer metastasis
Zhang Y et al. [318]	Inhibition	Fructose activates mTORC1 in adipocytes, inducing leptin, which enhances CD8^+^ T cell anti-tumor activity
Dewdney B et al. [319]	Inhibition	Fructose promotes apoptosis and inhibits proliferation of hepatoma cells

*SSP* the serine synthesis pathway, *DNL *de novo lipogenesis, *HO-1 *heme oxygenase-1, *LPCs *lysophosphatidylcholines, *AML *acute myeloid leukemia, *GLUT5 *glucose transporter 5, *AMPK *AMP-activated protein kinase, *KHK-A *ketohexokinase-A, *mTORC1 *mTOR Complex 1

Several intermediates produced during glycolysis can act as biomolecule synthesis precursors for the rapid expansion and metastasis of tumor cells. Tumor metabolism is highly plastic, enabling the utilization of available carbon sources to adapt to nutrient-stressed environments [157, 308, 309]. Abnormalities in glycolytic metabolism may disturb glucose levels in the tumor microenvironment. Fructose is a potential alternative carbon source that tumor cells use to maintain metabolism. After simple metabolism, fructose metabolites can directly enter glycolysis and bypass the key rate-limiting step of glycolytic phosphofructokinase to satisfy the demand for energy and biomolecule synthesis substrates in tumor cells and facilitate tumorigenesis and development. Under specific conditions, fructose can be phosphorylated to fructose 6-phosphate by hexokinase and directly enter glycolysis [16, 310]. Similar to glucose, fructose affects the survival, growth, and proliferation of tumor cells [311, 312].

#### The indirect tumor-promoting effect of fructose

Excessive fructose intake is associated with the development of gastrointestinal cancers and drives tumor growth and metastasis in mice with colorectal cancer [21, 22, 320–322]. Dietary fructose positively affects the survival and nutrient absorption of small intestinal cells in mice. Feeding mice with high-fructose syrup for four weeks increased the length of the small intestinal villi by 15–40%, increased the relative surface area of the intestine, improved nutrient absorption, and significantly increased body weight. The growth of mouse small intestinal villi is attributed to the extraction and metabolism of fructose by intestinal epithelial cells, which is rapidly converted to fructose 1-phosphate by KHK. In mice, fructose 1-phosphate was found to inhibit pyruvate kinase M2 activity—which protects intestinal epithelial cells—and promoted small intestinal epithelial cell survival, which ultimately increases intestinal tumor load [95]. A high-fructose diet upregulates heme oxygenase-1 (HO-1) expression, which makes mouse melanoma immune checkpoint inhibitor treatment resistant, and the use of HO-1 small-molecule inhibitors is effective in alleviating resistance [313].

In addition, fructose supplementation increases the amount of nutrients in the blood, which supports tumor development. According to a recently published study [314]. Because tumor cells lack the necessary enzymes to directly metabolize fructose, they are therefore less likely to use fructose for nutrition. The liver metabolizes most excess fructose. In co-culture studies, hepatocytes were found to have transformed fructose carbon into nutrients, which promoted the growth of cancer cells. The most noticeable alteration was observed in lysophosphatidylcholines (LPCs), in which cancer cells ingested and utilized phosphatidylcholine, the primary phospholipid found in cell membranes. Additionally, high-fructose corn syrup feeding in animal studies increases the number of LPC species in the blood of mice. These results imply that fructose indirectly stimulates tumor growth by increasing the levels of nutrients such as LPC in the blood.

Moreover, fructose may indirectly promote tumor growth by affecting the polarization of relevant immune cells. Fructose also promotes cancer cell growth by affecting the polarization of tumor-associated macrophages (TAMs) [315]. The interaction between hexokinase 2 and inositol-1,4,5-trisphosphate receptor 3 was improved by fructose treatment. This, in turn, affects intracellular calcium ion flow and signaling and finally inhibits the polarization of M1-type macrophages, which are known to have anti-tumor capabilities, thereby indirectly promoting the development of cancer.

#### The direct tumor-promoting effect of fructose

Some tumor cells upregulate the transcription of the GLUT5-encoding gene SLC2A5 to increase fructose utilization. Acute myeloid leukemia (AML) is metabolically characterized by SLC2A5-mediated high fructose utilization, which correlates with patient prognosis. The use of the small-molecule drug 2,5-anhydro-D-mannitol to block fructose transmembrane transport in a mouse AML model resulted in a significant improvement in leukemia symptoms and prolonged the survival of mice. The use of fructose transmembrane transport blockers in AML cells cultured in vitro were found to have significantly inhibited malignant proliferation and infiltration of cancer cells [316]. Glioma cell lines show high levels of GLUT5 expression, and the upregulation of GLUT5 expression in the glioma tissues of patients is usually associated with poor prognosis. Under glucose-poor culture conditions, the survival and proliferation of glioma cells (in vitro cultures) were found to be promoted by upregulating GLUT5 expression [323]. The glioma cell line LN229 was used to study subcutaneous tumor formation in mice fed 15% fructose water. The results showed that mice administered fructose water had larger tumor volumes and smaller foci of tumor necrosis. However, the knockdown of GLUT5 in LN229 cells was followed by the inoculation of mice with tumors, and mice in the fructose-fed group did not show any of these conditions [323]. The authors of the study reported that the analysis of clinical samples revealed that the expression of the fructose transporter proteins GLUT5 and GLUT9 was upregulated in patients with prostate cancer, and that their serum fructose levels were higher than those in normal subjects. In another study, fructose treatment of in vitro cultured prostate cancer cells PC3 revealed that fructose promoted the proliferation and invasion of prostate cancer cells. Based on the transcriptome analysis of the PC3 cell line, fructose activates the proliferation-related pathway of prostate cancer cells and up-regulates the expression of transforming growth factor-β, wingless-type MMTV integration site family-4, and other genes; the authors concluded that fructose-fed prostate cancer cell xenograft tumor model mice promotes prostate tumor growth and proliferation [324]. In addition, fructose accelerates lung cancer cell growth in vivo by upregulating GLUT5 protein expression and inhibiting AMPK, which activates mTORC1 activity and promotes fatty acid synthesis and palmitoleic acid production [325].

KHK, a key enzyme in fructose metabolism, is involved in fructose utilization by glioma cells and promotes tumor progression. Analysis of clinical samples revealed that high KHK expression was associated with poor prognosis. Silencing KHK in glioma cells significantly inhibited their proliferation and migration. Glioma cells cultured in fructose medium for four weeks show upregulated *KHK* gene expression, increased protein stability, and upregulated KHK expression, which accelerated the malignant progression of tumors [326].

In primary hepatocellular carcinoma, the downregulation of aldolase B expression correlates with increased tumor aggressiveness and is usually associated with poor prognosis. Stable expression of aldolase B in primary hepatocellular carcinoma promotes the expression of DNA demethylase Ten-Eleven Translocation 1, which reduces the migratory ability of hepatocellular carcinoma cells in vitro and their metastatic potential in vivo [327]. In contrast, in colorectal cancer liver metastasis, aldolase B expression is upregulated and fructose metabolism is enhanced, allowing colorectal cancer cells to rapidly adapt to the high-fructose environment of the liver. Enhanced fructose metabolism increases gluconeogenesis, glycolysis, and the pentose phosphate pathway, which provides the corresponding substrates for central carbon metabolism during tumor proliferation and promotes the growth of metastatic liver tumors from colorectal cancer. Targeting aldolase B or its upstream regulator GATA6 may be effective, and reducing fructose intake is important for controlling liver metastasis [22].

In a study by Goncalves et al. an intestinal tumorigenesis model using adenomatous polyposis coli mutant mice (APC^−/−^ mice) that were administered by gavage high-fructose syrup water equivalent to the daily dose of one can of soda consumed by a human. Eight weeks later, APC^−/−^ mice did not show a significant increase in body weight, but they had an increased tumor load. This is most likely because daily intake of high-fructose syrup creates a high-fructose environment in the intestinal lumen of mice. Excessive fructose is metabolized through the consumption of a large amount of ATP, and low levels of ATP activate phosphofructokinase, which increases glucose metabolism flux and directs fatty acid synthesis. Fatty acids are important for tumor growth and are biomolecular substrates used by tumor cells to synthesize cytosolic or signaling molecules. The authors of the study concluded that fructose promotes intestinal tumorigenesis in mice by promoting glycolysis and DNL [21].

Serine is an essential nutrient specific to tumor cells, and the serine synthesis pathway (SSP) is important for tumor metabolism. In SSP, 3-phosphoglycerol derived from glycolysis is converted to serine by various enzymes to provide the metabolic precursors for one-carbon metabolism [328]. Meanwhile, α-ketoglutarate produced during SSP enters the TCA cycle, providing substrates for nucleotide synthesis and to maintain redox balance among tumor cells [328–332]. High fructose levels can drive SSP in AML cells and exacerbate tumor burden. Acute myeloid leukemia cells are more SSP-dependent in high-fructose environments. These cells mediate their proliferation in the presence of glucose deficiency by upregulating SSP flux and producing α-ketoglutarate from glutamine. Targeting the rate-limiting enzyme phosphoglycerol dehydrogenase in SSP in a high-fructose environment significantly reduced tumor load and slowed leukemia progression in mice [16]. Moreover, fructose promotes mitochondrial respiration by activating AMPK, which increases the proliferation and migration capacity of tumor endothelial cells and promotes tumor angiogenesis, growth, and metastasis in hepatocellular carcinoma xenografts and Myc/sgp53-induced hepatocellular carcinoma mouse models [298]. An SLC2A5 inhibitor was hsown to have effectively inhibited fructose-induced tumor angiogenesis and suppresses tumor growth in mice [298].

The polyol pathway facilitates the production of endogenous fructose. Glucose is reduced to sorbitol by NADPH and aldose reductase. Sorbitol is oxidized by NAD^+^ and catalyzed by sorbitol dehydrogenase to produce endogenous fructose and NADH [333]. Endogenous fructose production coupled with KHK, which is commonly highly expressed in tumors, bypasses the rate-limiting step in glycolysis and rapidly meets the energy and substrate requirements of tumor cell growth, thereby promoting tumor cell growth. Schwab et al. reported a direct correlation between the polyol pathway activity and tumor development [334]. Aldo–keto reductase family 1 member B1 (AKR1B1) encodes a specific member of the Aldo–keto reductase superfamily that catalyzes the reduction of glucose to sorbitol and has an important role in the polyol pathway [335]. AKR1BA is associated with epithelial mesenchymal transition in lung cancer patient samples. Inhibition of epithelial-to-mesenchymal transition was found to occur after in vitro knockdown of AKR1B1 in mesenchymal-like cancer cells [334]. There is a link between the polyol pathway and gastric cancer progression. In gastric cancer cell lines, hyperglycemia induces endogenous fructose formation by increasing the flux of the polyol pathway, which in turn activates the KHK-A signaling pathway and inhibits CDH1 expression, thus inducing epithelial mesenchymal transition and promoting gastric cancer metastasis [317].

When taken together, fructose induces metabolic stress in tumor cells, leading to metabolic reprogramming. Fructose promotes tumor development mainly by upregulating the expression of the fructose transporter, GLUT5, and metabolism-related enzymes to meet the energy and substrate requirements of tumors. The metabolic reprogramming of tumor cells increases fructose metabolism, glycolysis, DNL, and polyol pathway fluxes through multiple pathways, providing synthetic precursors for tumor development, regulating functional gene expression, and promoting tumor progression [336, 337]. Thus, the fructose transporter protein GLUT5 or the key enzymes of fructose metabolism, KHK and aldolase B, are potential targets for anticancer treatment.

#### The tumor-suppressing effect of fructose

Although the mainstream view among researchers is that fructose promotes cancer, some epidemiological studies have shown that fructose is protective against oral and lung cancers in men, and against lung and ovarian cancers in women [318, 321, 338]. Recently, Zou et al. published their latest research on fructose and tumors [318]. In contrast to the generally held view, the authors reported that that fructose inhibited tumor growth and induced leptin secretion from adipocytes by activating mTORC1 in white adipose tissue cells. Adipocyte-derived leptin acts on CD8^+^ T cells to enhance the anti-tumor activity of CD8^+^ T cells to control transplanted lung tumors. Transcriptome analysis of CD8^+^ T cells revealed that tumor-infiltrating CD8^+^ T cells in high-fructose-fed mice were predominantly early stage CD8^+^ T cells, whereas tumor-infiltrating CD8^+^ T cells in fructose-free control mice were predominantly exhausted. Therefore, fructose treatment can reduce the number of exhausted CD8^+^ T cells, downregulate the overall exhaustion rate of CD8^+^ T cells, and increase the proliferation rate and IFN-γ production of CD8^+^ T cells, thereby achieving anti-tumor effects (Fig. 4) [318]. Currently, it is not clear how fructose and leptin inhibit CD8^+^ T cell exhaustion. One possible mechanism is that fructose and leptin reorganize T cell metabolism and maintain T cell stemness. Moreover, the in-depth mechanism of the fructose-leptin axis in the regulation of the tumor immune microenvironment and its application in tumor immunotherapy warrant further study. In addition to fighting tumors by regulating the immune microenvironment, fructose was found to significantly inhibit the proliferation of cultured hepatoma cells Huh7 and promote cell apoptosis [319], indicating that fructose may have a direct anti-tumor function in some tumors.

Given the positive role of fructose in CD8^+^ T cell-mediated antitumor immunity, the use of fructose as a nutritional supplement or in combination of fructose supplementation with adoptive T cell therapy may be a promising avenue for cancer treatment. However, further clinical evidence is still required to determine whether this effect occurs in humans. In addition to affecting adipocyte metabolism, the direct role of fructose in regulating T-cell metabolism and function has not yet been identified. When considering the opposing effects of fructose on tumors, fructose may exert different regulatory effects on different tumors. Therefore, the complex regulatory mechanisms of fructose in specific tumors and the tumor immune microenvironment require further investigation.

## Therapeutic modulation of fructose metabolism

In modern society, human consumption of fructose is increasing, and the adverse effects of fructose on humans are becoming increasingly clear. Today, regulating the metabolism of fructose has become a key area in the investigation of metabolic diseases and inflammation, and is a highly promising field for cancer treatment.

### Intervention of fructose metabolism through the GLUT5-KHK axis

GLUT5 expression is linked with numerous metabolic syndromes and tumors. GLUT5 inhibition lowers the total fructose in the body, which may have beneficial implications in slowing metabolic disorders, reducing inflammation, and even interfering with tumor development [339, 340]. Only a few GLUT5 inhibitors are currently available; one study yielded a small molecule inhibitor, N-[4-(methylsulfonyl)−2-nitrophenyl]−1,3-benzodioxol-5-amine (MSNBA), which blocks fructose uptake by specifically binding to the fructose site of GLUT5 [341]. Identifying it may aid in studying fructose and its related health issues.

KHK is a crucial enzyme in the metabolism of fructose and the first to act on it. KHK catalyzes the conversion of fructose to fructose 1-phosphate, and hence is a very vital target for controlling the metabolism of fructose. KHK inhibition markedly decreases hepatic fat deposition, enhances glucose tolerance, and reverses MAFLD [342–344]. Recently, the synthesis of the inhibitors of KHK has been accomplished; one such inhibitor, PF-06835919, has drawn much interest [345]. In a phase 2 randomized clinical trial, 300 mg of PF-06835919 reduced the whole liver fat mass in MAFLD patients and inflammatory markers. This offers a novel therapeutic approach for managing metabolic disorders caused by fructose and inflammatory reactions [346]. Other efficient KHK inhibitors have also been reported; however, clinical studies remain warranted to test these drugs [347]. KHK expression in several tumor cells is abnormally high, and fructose can drive tumor growth and metastasis through the activity of KHK [297, 310, 348]. This suggests that targeting KHK may be a potential cancer treatment strategy.

### Intervention of fructose metabolism through modulation of gut microbes

Fructose is directly linked to gut flora dysbiosis. Fructose feeding has been shown to modify the gut flora composition to induce an inflammatory response and metabolic abnormalities; regulating the gut microbiota to correct fructose metabolism may counteract fructose-induced illness [349–351]. Broad-spectrum antibiotics can suppress the hippocampal neuro-inflammation in fructose-fed mice by regulating gut microbiota; however, the effectiveness of antibiotics needs to be verified through more definitive studies, considering their safe use in humans [192]. Certain natural antioxidants isolated from plants and animals have therapeutic properties. For instance, the water extract of *Lycium ruthenicum* Murray ameliorates neuroinflammation and cognitive deficits induced by a high-fructose diet by modifying the gut-liver-brain axis. Furthermore, it can alter the composition of the intestinal microbiota, increasing the abundance of beneficial bacteria [352]. Anthocyanins from *Lycium ruthenicum* Murray can effectively reduce the ecological dysbiosis of the intestinal microbiota and preserve the integrity of the intestinal barrier, lowering neuroinflammation caused by a high-fructose diet in rats [353].

Individuals seem to respond more readily to the therapeutic advantages of these bioactive compounds than to antibiotics. These substances exhibit significant biological activities, particularly antioxidant activities, and have numerous health benefits, in addition to their role in treating fructose-induced disorders. Furthermore, most of these compounds are safe and nontoxic; therefore, developing therapeutic drugs based on these biologically active substances is promising. Current research on reversing and preventing the side effects of fructose is still limited because of unclear mechanisms and the complexity of its effects, particularly in targeted therapy.

## Conclusion and future perspectives

Fructose, the sweetest hexose in nature, is a popular additive in processed foods and beverages, the metabolism of which plays a key role in the response of organisms to extreme environmental conditions (such as food, water, and oxygen shortages). Fructose is metabolized differently from glucose, leading to its rapid absorption in the liver. However, high consumption of fructose is frequently linked to obesity and several metabolic disorders, particularly in nations experiencing greater economic development and greater access to processed foods [7]. The relationship between fructose and obesity remains debatable: some studies suggest that fructose directly causes obesity, whereas others indicate that fructose does not appear to have a direct impact on obesity [86, 92]. The intricacy of fructose metabolism in the body is reflected in this side, and further research is necessary to determine whether fructose induces obesity directly. Also, the triggers of metabolic diseases are extremely complex and involve various biological processes, such as dysregulation of adipose synthesis, activation of specific cellular receptors, and aberrant expression of related pathways (Fig. 2). Although several studies have revealed that fructose can induce a wide range of metabolic diseases, including MAFLD, hyperinsulinemia, hyperuricemia, and hypertension, the underlying mechanisms remain obscure and require further investigations. Because of different experimental conditions and testing standards, conflicting views exist among researchers [26, 354–357]; therefore, when investigating the connection between metabolic diseases and fructose consumption, the association must be confirmed from various aspects.

Moreover, inflammation is a detrimental effect of fructose, with substantial evidence indicating that excessive fructose intake drives inflammatory effects via various mechanisms (Fig. 3). In the intestine, fructose damages the intestinal barrier and causes dysbiosis of the intestinal bacterial flora, directly or indirectly, inducing inflammatory responses [201, 206]. When fructose enters the liver, it induces ER and oxidative stress in liver cells, activating relevant inflammatory pathways [232]. Macrophages in the liver are negatively affected by fructose. Indeed, excessive fructose intake induces an inflammatory response in the brain, affecting the hippocampus, hypothalamus, and parts of the cerebral cortex. Additionally, fructose induces a systemic inflammatory response, and its harmful effects cannot be ignored. Therefore, future research should focus on the latent targets of these induction mechanisms to identify relevant therapeutic approaches.

Fructose increases tumor risk (Table 4). Tumor cells exhibit abnormally high glycolytic metabolic activity and rapidly consume glucose [309]. Fructose is the second most abundant blood sugar in the body, and a high-quality alternative carbon source for tumor cells to support survival and growth when glucose is scarce [310]. Fructose provides energy for tumor cells and biomolecular precursors for tumor cell proliferation by increasing the flux of glycolysis, the PPP, and the polyol pathway. Even if tumor cells do not directly metabolize fructose, they still produce large amounts of circulating nutrients through liver cells to promote tumor growth. In turn, fructose affects anti-tumor-related immune cells to promote tumor development.

However, a recent study has confirmed that fructose promotes cancer by demonstrating that fructose enhances the anti-tumor response of CD8^+^ T cells by promoting leptin secretion from adipocytes (Fig. 4) [318]. Currently, it appears that the effect of fructose on tumor cells is a “double-edged sword.” Research on the role of fructose should be based on the specific tumor microenvironment involved in fructose metabolism, tumor metabolism, and other complex and precise regulatory mechanisms.

Multilevel targeted fructose metabolism or targeted fructose metabolism in combination with current chemotherapeutic and immunosuppressive drugs may provide novel cancer treatments. Current research on the role of fructose in tumors tends to focus on metabolic changes in tumor cells in a high-fructose environment, whereas research on the influence of fructose on immune cells in the tumor microenvironment is scarce. Future research should explore the metabolic and behavioral changes in immune cells in the tumor microenvironment in a high-fructose environment. Several studies investigating the effects of fructose on the immune cells indicate that fructose induces metabolic reprogramming of multiple immune cells and increases immune cell inflammation. Whether future cell therapies can specifically target immune cells to deliver fructose and enhance the anti-tumor activity of immune cells remains to be determined.

Preliminary studies on intervention strategies for fructose-induced diseases have been conducted. GLUT5 is the primary fructose transporter. Although targeting it has therapeutic potential, GLUT5 has several physiological roles; therefore, side effects associated with blocking its expression must be considered. The effectiveness of PF-06835919, a small-molecule inhibitor of KHK, has been demonstrated in clinical trials, the results of which confirmed the therapeutic utility in targeting KHK [358]. However, KHK with aberrantly high-level expression in cancer is KHK-A and not KHK-C, indicating that the development of KHK inhibitors must address the problem of selectivity to mitigate side effects [310]. In regulating gut microbiota, the exploration and application of biologically active substances have attracted interest. These substances might offer numerous undiscovered advantages for humans, with alleviating fructose-induced diseases being just one of many effects. Therefore, this class of substances has great potential for future drug development. In addition, substances that modulate the gut microbiota (e.g., probiotics and prebiotics) may have similar effects and should, therefore, be explored [11]. Interventions targeting the gut microbiome may need to consider differences among various populations. [349]. Overall, intervention via fructose metabolism requires targeting multiple pathways and individualized approaches. However, more simply, reducing fructose intake may be the most straightforward strategy.

## Data Availability

Not applicable.

## References

[1] Johnson RJ, Stenvinkel P, Andrews P, Sánchez-Lozada LG, Nakagawa T, Gaucher E, et al. Fructose metabolism as a common evolutionary pathway of survival associated with climate change, food shortage and droughts. J Intern Med. 2020;287(3):252–62. 10.1111/joim.12993. 31621967 10.1111/joim.12993 PMC10917390

[2] Tappy L, Lê KA. Metabolic effects of fructose and the worldwide increase in obesity. Physiol Rev. 2010;90(1):23–46. 10.1152/physrev.00019.2009. 20086073 10.1152/physrev.00019.2009

[3] Rippe JM, Angelopoulos TJ. Fructose-containing sugars and cardiovascular disease. Adv Nutr. 2015;6(4):430–9. 10.3945/an.114.008177. 26178027 10.3945/an.114.008177 PMC4496738

[4] Righetti S, Medoro A, Graziano F, Mondazzi L, Martegani S, Chiappero F, et al. Effects of Maltodextrin-Fructose Supplementation on Inflammatory Biomarkers and Lipidomic Profile Following Endurance Running: A Randomized Placebo-Controlled Cross-Over Trial. Nutrients. 2024;16(18). 10.3390/nu16183078. 10.3390/nu16183078 PMC11434986 39339678

[5] Semchyshyn H. Fructose-mediated AGE-RAGE axis: approaches for mild modulation. Front Nutr. 2024;11:1500375. 10.3389/fnut.2024.1500375. 39698244 10.3389/fnut.2024.1500375 PMC11652219

[6] Livesey G, Taylor R. Fructose consumption and consequences for glycation, plasma triacylglycerol, and body weight: meta-analyses and meta-regression models of intervention studies. Am J Clin Nutr. 2008;88(5):1419–37. 10.3945/ajcn.2007.25700. 18996880 10.3945/ajcn.2007.25700

[7] Febbraio MA, Karin M. “Sweet death”: Fructose as a metabolic toxin that targets the gut-liver axis. Cell Metab. 2021;33(12):2316–28. 10.1016/j.cmet.2021.09.004. 34619076 10.1016/j.cmet.2021.09.004 PMC8665123

[8] Bray GA, Nielsen SJ, Popkin BM. Consumption of high-fructose corn syrup in beverages may play a role in the epidemic of obesity. Am J Clin Nutr. 2004;79(4):537–43. 10.1093/ajcn/79.4.537. 15051594 10.1093/ajcn/79.4.537

[9] Lanaspa MA, Sanchez-Lozada LG, Cicerchi C, Li N, Roncal-Jimenez CA, Ishimoto T, et al. Uric acid stimulates fructokinase and accelerates fructose metabolism in the development of fatty liver. PLoS One. 2012;7(10):e47948. 10.1371/journal.pone.0047948. 23112875 10.1371/journal.pone.0047948 PMC3480441

[10] Lodge M, Dykes R, Kennedy A. Regulation of Fructose Metabolism in Nonalcoholic Fatty Liver Disease. Biomolecules. 2024;14(7). 10.3390/biom14070845. 10.3390/biom14070845 PMC11274671 39062559

[11] Jung S, Bae H, Song WS, Jang C. Dietary Fructose and Fructose-Induced Pathologies. Annu Rev Nutr. 2022;42:45–66. 10.1146/annurev-nutr-062220-025831. 35995049 10.1146/annurev-nutr-062220-025831 PMC9904196

[12] Jaiswal N, Agrawal S, Agrawal A. High fructose-induced metabolic changes enhance inflammation in human dendritic cells. Clin Exp Immunol. 2019;197(2):237–49. 10.1111/cei.13299. 30919933 10.1111/cei.13299 PMC6642875

[13] Jones N, Blagih J, Zani F, Rees A, Hill DG, Jenkins BJ, et al. Fructose reprogrammes glutamine-dependent oxidative metabolism to support LPS-induced inflammation. Nat Commun. 2021;12(1):1209. 10.1038/s41467-021-21461-4. 33619282 10.1038/s41467-021-21461-4 PMC7900179

[14] Wagnerberger S, Spruss A, Kanuri G, Volynets V, Stahl C, Bischoff SC, et al. Toll-like receptors 1–9 are elevated in livers with fructose-induced hepatic steatosis. Br J Nutr. 2012;107(12):1727–38. 10.1017/s0007114511004983. 22018861 10.1017/S0007114511004983

[15] Dewdney B, Roberts A, Qiao L, George J, Hebbard L. A Sweet Connection? Fructose's Role in Hepatocellular Carcinoma. Biomolecules. 2020;10(4). 10.3390/biom10040496. 10.3390/biom10040496 PMC7226025 32218179

[16] Jeong S, Savino AM, Chirayil R, Barin E, Cheng Y, Park SM, et al. High Fructose Drives the Serine Synthesis Pathway in Acute Myeloid Leukemic Cells. Cell Metab. 2021;33(1):145-59.e6. 10.1016/j.cmet.2020.12.005. 33357456 10.1016/j.cmet.2020.12.005 PMC8168776

[17] Taskinen MR, Packard CJ, Borén J. Dietary Fructose and the Metabolic Syndrome. Nutrients. 2019;11(9). 10.3390/nu11091987. 10.3390/nu11091987 PMC6770027 31443567

[18] Mirtschink P, Jang C, Arany Z, Krek W. Fructose metabolism, cardiometabolic risk, and the epidemic of coronary artery disease. Eur Heart J. 2018;39(26):2497–505. 10.1093/eurheartj/ehx518. 29020416 10.1093/eurheartj/ehx518 PMC6037111

[19] Herman MA, Birnbaum MJ. Molecular aspects of fructose metabolism and metabolic disease. Cell Metab. 2021;33(12):2329–54. 10.1016/j.cmet.2021.09.010. 34619074 10.1016/j.cmet.2021.09.010 PMC8665132

[20] Wang Y, Qi W, Song G, Pang S, Peng Z, Li Y, et al. High-Fructose Diet Increases Inflammatory Cytokines and Alters Gut Microbiota Composition in Rats. Mediators Inflamm. 2020;2020:6672636. 10.1155/2020/6672636. 33312070 10.1155/2020/6672636 PMC7721508

[21] Goncalves MD, Lu C, Tutnauer J, Hartman TE, Hwang SK, Murphy CJ, et al. High-fructose corn syrup enhances intestinal tumor growth in mice. Science. 2019;363(6433):1345–9. 10.1126/science.aat8515. 30898933 10.1126/science.aat8515 PMC6487857

[22] Bu P, Chen KY, Xiang K, Johnson C, Crown SB, Rakhilin N, et al. Aldolase B-Mediated Fructose Metabolism Drives Metabolic Reprogramming of Colon Cancer Liver Metastasis. Cell Metab. 2018;27(6):1249-62.e4. 10.1016/j.cmet.2018.04.003. 29706565 10.1016/j.cmet.2018.04.003 PMC5990465

[23] Liu C, Wo J, Zhao Q, Wang Y, Wang B, Zhao W. Association between Serum Total Osteocalcin Level and Type 2 Diabetes Mellitus: A Systematic Review and Meta-Analysis. Horm Metab Res. 2015;47(11):813–9. 10.1055/s-0035-1564134. 26372899 10.1055/s-0035-1564134

[24] Abdelmalek MF, Suzuki A, Guy C, Unalp-Arida A, Colvin R, Johnson RJ, et al. Increased fructose consumption is associated with fibrosis severity in patients with nonalcoholic fatty liver disease. Hepatology. 2010;51(6):1961–71. 10.1002/hep.23535. 20301112 10.1002/hep.23535 PMC2922495

[25] Ouchi Y, Komaki Y, Shimizu K, Fukano N, Sugino T, Shiraishi JI, et al. Comparison of oral administration of fructose and glucose on food intake and physiological parameters in broiler chicks. Poult Sci. 2023;102(1):102249. 10.1016/j.psj.2022.102249. 36335736 10.1016/j.psj.2022.102249 PMC9640322

[26] Le MT, Frye RF, Rivard CJ, Cheng J, McFann KK, Segal MS, et al. Effects of high-fructose corn syrup and sucrose on the pharmacokinetics of fructose and acute metabolic and hemodynamic responses in healthy subjects. Metabolism. 2012;61(5):641–51. 10.1016/j.metabol.2011.09.013. 22152650 10.1016/j.metabol.2011.09.013 PMC3306467

[27] Tran C, Jacot-Descombes D, Lecoultre V, Fielding BA, Carrel G, Lê KA, et al. Sex differences in lipid and glucose kinetics after ingestion of an acute oral fructose load. Br J Nutr. 2010;104(8):1139–47. 10.1017/s000711451000190x. 20540820 10.1017/S000711451000190X

[28] Teff KL, Grudziak J, Townsend RR, Dunn TN, Grant RW, Adams SH, et al. Endocrine and Metabolic Effects of Consuming Fructose- and Glucose-Sweetened Beverages with Meals in Obese Men and Women: Influence of Insulin Resistance on Plasma Triglyceride Responses. Journal of Clinical Endocrinology & Metabolism. 2009;94(5):1562–9. 10.1210/jc.2008-2192. 19208729 10.1210/jc.2008-2192 PMC2684484

[29] Sugimoto K, Kawasaki T, Tomoda M, Nakagawa K, Hayashi S, Inui H, et al. Lowering of Postprandial Hyperfructosemia in Humans by Eucalyptus Leaf Extract: A Randomized, Double-blind, Placebo-controlled Crossover Study. Food Science and Technology Research. 2010;16(5):509–12. 10.3136/fstr.16.509.

[30] Wahjudi PN, Patterson ME, Lim S, Yee JK, Mao CS, Lee WN. Measurement of glucose and fructose in clinical samples using gas chromatography/mass spectrometry. Clin Biochem. 2010;43(1–2):198–207. 10.1016/j.clinbiochem.2009.08.028. 19747474 10.1016/j.clinbiochem.2009.08.028 PMC2812637

[31] Preston GM, Calle RA. Elevated Serum Sorbitol and not Fructose in Type 2 Diabetic Patients. Biomark Insights. 2010;5:33–8. 10.4137/bmi.s4530. 20520742 10.4137/bmi.s4530 PMC2879225

[32] Jang C, Hui S, Lu W, Cowan AJ, Morscher RJ, Lee G, et al. The Small Intestine Converts Dietary Fructose into Glucose and Organic Acids. Cell Metab. 2018;27(2):351-61.e3. 10.1016/j.cmet.2017.12.016. 29414685 10.1016/j.cmet.2017.12.016 PMC6032988

[33] Vos MB, Lavine JE. Dietary fructose in nonalcoholic fatty liver disease. Hepatology. 2013;57(6):2525–31. 10.1002/hep.26299. 23390127 10.1002/hep.26299

[34] Barone S, Fussell SL, Singh AK, Lucas F, Xu J, Kim C, et al. Slc2a5 (Glut5) is essential for the absorption of fructose in the intestine and generation of fructose-induced hypertension. J Biol Chem. 2009;284(8):5056–66. 10.1074/jbc.M808128200. 19091748 10.1074/jbc.M808128200 PMC2643499

[35] Wood IS, Trayhurn P. Glucose transporters (GLUT and SGLT): expanded families of sugar transport proteins. Br J Nutr. 2003;89(1):3–9. 10.1079/bjn2002763. 12568659 10.1079/BJN2002763

[36] Karim S, Adams DH, Lalor PF. Hepatic expression and cellular distribution of the glucose transporter family. World J Gastroenterol. 2012;18(46):6771–81. 10.3748/wjg.v18.i46.6771. 23239915 10.3748/wjg.v18.i46.6771 PMC3520166

[37] DeBosch BJ, Chi M, Moley KH. Glucose transporter 8 (GLUT8) regulates enterocyte fructose transport and global mammalian fructose utilization. Endocrinology. 2012;153(9):4181–91. 10.1210/en.2012-1541. 22822162 10.1210/en.2012-1541 PMC3423610

[38] Schmidt S, Joost HG, Schürmann A. GLUT8, the enigmatic intracellular hexose transporter. Am J Physiol Endocrinol Metab. 2009;296(4):E614-8. 10.1152/ajpendo.91019.2008. 19176349 10.1152/ajpendo.91019.2008

[39] Bismut H, Hers HG, Van Schaftingen E. Conversion of fructose to glucose in the rabbit small intestine A reappraisal of the direct pathway. Eur J Biochem. 1993;213(2):721–6. 10.1111/j.1432-1033.1993.tb17812.x. 8477744 10.1111/j.1432-1033.1993.tb17812.x

[40] Cheng H, Zhou J, Sun Y, Zhan Q, Zhang D. High fructose diet: A risk factor for immune system dysregulation. Hum Immunol. 2022;83(6):538–46. 10.1016/j.humimm.2022.03.007. 35414462 10.1016/j.humimm.2022.03.007

[41] Rumessen JJ. Fructose and related food carbohydrates Sources intake absorption and clinical implications. Scand J Gastroenterol. 1992;27(10):819–28. 10.3109/00365529209000148. 1439534 10.3109/00365529209000148

[42] Mavrias DA, Mayer RJ. Metabolism of fructose in the small intestine 1 The effect of fructose feeding on fructose transport and metabolism in rat small intestine. Biochim Biophys Acta. 1973;291(2):531–7. 10.1016/0005-2736(73)90504-x. 4690865 10.1016/0005-2736(73)90504-x

[43] Ginsburg V, Hers HG. On the conversion of fructose to glucose by guinea pig intestine. Biochim Biophys Acta. 1960;38:427–34. 10.1016/0006-3002(60)91278-6. 13850248 10.1016/0006-3002(60)91278-6

[44] Tappy L, Lê KA. Does fructose consumption contribute to non-alcoholic fatty liver disease? Clin Res Hepatol Gastroenterol. 2012;36(6):554–60. 10.1016/j.clinre.2012.06.005. 22795319 10.1016/j.clinre.2012.06.005

[45] Hannou SA, Haslam DE, McKeown NM, Herman MA. Fructose metabolism and metabolic disease. J Clin Invest. 2018;128(2):545–55. 10.1172/jci96702. 29388924 10.1172/JCI96702 PMC5785258

[46] Ferraris RP, Choe J-y, Patel CR. Intestinal Absorption of Fructose. Ann Rev Nutri. 2018;38(Volume 38, 2018):41–67. 10.1146/annurev-nutr-082117-051707. 10.1146/annurev-nutr-082117-051707 PMC6457363 29751733

[47] Patel C, Douard V, Yu S, Tharabenjasin P, Gao N, Ferraris RP. Fructose-induced increases in expression of intestinal fructolytic and gluconeogenic genes are regulated by GLUT5 and KHK. Am J Physiol Regul Integr Comp Physiol. 2015;309(5):R499-509. 10.1152/ajpregu.00128.2015. 26084694 10.1152/ajpregu.00128.2015 PMC4591376

[48] Ebert K, Ludwig M, Geillinger KE, Schoberth GC, Essenwanger J, Stolz J, et al. Reassessment of GLUT7 and GLUT9 as Putative Fructose and Glucose Transporters. J Membr Biol. 2017;250(2):171–82. 10.1007/s00232-016-9945-7. 28083649 10.1007/s00232-016-9945-7

[49] Gan Q, Song G, Fang W, Wang Y, Qi W. Fructose dose-dependently influences colon barrier function by regulation of some main physical, immune, and biological factors in rats. J Nutr Biochem. 2024;126:109582. 10.1016/j.jnutbio.2024.109582. 38242179 10.1016/j.jnutbio.2024.109582

[50] Koene E, Schrauwen-Hinderling VB, Schrauwen P, Brouwers M. Novel insights in intestinal and hepatic fructose metabolism: from mice to men. Curr Opin Clin Nutr Metab Care. 2022;25(5):354–9. 10.1097/mco.0000000000000853. 35838297 10.1097/MCO.0000000000000853

[51] Salomon LL, Lanza FL, Smith DE. Renal conversion of fructose to glucose. Am J Physiol. 1961;200:871–7. 10.1152/ajplegacy.1961.200.4.871. 13745722 10.1152/ajplegacy.1961.200.4.871

[52] Björkman O, Felig P. Role of the kidney in the metabolism of fructose in 60-hour fasted humans. Diabetes. 1982;31(6 Pt 1):516–20. 10.2337/diab.31.6.516. 6130022 10.2337/diab.31.6.516

[53] Burch HB, Choi S, Dence CN, Alvey TR, Cole BR, Lowry OH. Metabolic effects of large fructose loads in different parts of the rat nephron. J Biol Chem. 1980;255(17):8239–44. 6773936

[54] Nakagawa T, Johnson RJ, Andres-Hernando A, Roncal-Jimenez C, Sanchez-Lozada LG, Tolan DR, et al. Fructose Production and Metabolism in the Kidney. J Am Soc Nephrol. 2020;31(5):898–906. 10.1681/asn.2019101015. 32253274 10.1681/ASN.2019101015 PMC7217403

[55] Grempler R, Augustin R, Froehner S, Hildebrandt T, Simon E, Mark M, et al. Functional characterisation of human SGLT-5 as a novel kidney-specific sodium-dependent sugar transporter. FEBS Lett. 2012;586(3):248–53. 10.1016/j.febslet.2011.12.027. 22212718 10.1016/j.febslet.2011.12.027

[56] Fukuzawa T, Fukazawa M, Ueda O, Shimada H, Kito A, Kakefuda M, et al. SGLT5 reabsorbs fructose in the kidney but its deficiency paradoxically exacerbates hepatic steatosis induced by fructose. PLoS One. 2013;8(2):e56681. 10.1371/journal.pone.0056681. 23451068 10.1371/journal.pone.0056681 PMC3581502

[57] Gonzalez-Vicente A, Cabral PD, Hong NJ, Asirwatham J, Saez F, Garvin JL. Fructose reabsorption by rat proximal tubules: role of Na(+)-linked cotransporters and the effect of dietary fructose. Am J Physiol Renal Physiol. 2019;316(3):F473-f80. 10.1152/ajprenal.00247.2018. 30565998 10.1152/ajprenal.00247.2018 PMC6459303

[58] Kawasaki T, Akanuma H, Yamanouchi T. Increased fructose concentrations in blood and urine in patients with diabetes. Diabetes Care. 2002;25(2):353–7. 10.2337/diacare.25.2.353. 11815509 10.2337/diacare.25.2.353

[59] Vallon V, Nakagawa T. Renal Tubular Handling of Glucose and Fructose in Health and Disease. Compr Physiol. 2021;12(1):2995–3044. 10.1002/cphy.c210030. 34964123 10.1002/cphy.c210030 PMC9832976

[60] Froesch ER, Ginsberg JL. Fructose metabolism of adipose tissue I Comparison of fructose and glucose metabolism in epididymal adipose tissue of normal rats. J Biol Chem. 1962;237:3317–24. 13959931

[61] Zubiría MG, Alzamendi A, Moreno G, Rey MA, Spinedi E, Giovambattista A. Long-Term Fructose Intake Increases Adipogenic Potential: Evidence of Direct Effects of Fructose on Adipocyte Precursor Cells. Nutrients. 2016;8(4):198. 10.3390/nu8040198. 27049396 10.3390/nu8040198 PMC4848667

[62] Ahlborg G, Björkman O. Splanchnic and muscle fructose metabolism during and after exercise. J Appl Physiol (1985). 1990;69(4):1244–51. 10.1152/jappl.1990.69.4.1244. 2262441 10.1152/jappl.1990.69.4.1244

[63] Hajduch E, Darakhshan F, Hundal HS. Fructose uptake in rat adipocytes: GLUT5 expression and the effects of streptozotocin-induced diabetes. Diabetologia. 1998;41(7):821–8. 10.1007/s001250050993. 9686924 10.1007/s001250050993

[64] Helsley RN, Moreau F, Gupta MK, Radulescu A, DeBosch B, Softic S. Tissue-Specific Fructose Metabolism in Obesity and Diabetes. Curr Diab Rep. 2020;20(11):64. 10.1007/s11892-020-01342-8. 33057854 10.1007/s11892-020-01342-8 PMC10208418

[65] Viegas I, Di Nunzio G, Belew GD, Torres AN, Silva JG, Perpétuo L, et al. Integration of Liver Glycogen and Triglyceride NMR Isotopomer Analyses Provides a Comprehensive Coverage of Hepatic Glucose and Fructose Metabolism. Metabolites. 2022;12(11). 10.3390/metabo12111142. 10.3390/metabo12111142 PMC9698123 36422282

[66] Lodge M, Scheidemantle G, Adams VR, Cottam MA, Richard D, Breuer D, et al. Fructose regulates the pentose phosphate pathway and induces an inflammatory and resolution phenotype in Kupffer cells. Sci Rep. 2024;14(1):4020. 10.1038/s41598-024-54272-w. 38369593 10.1038/s41598-024-54272-w PMC10874942

[67] Sahoo B, Srivastava M, Katiyar A, Ecelbarger C, Tiwari S. Liver or kidney: Who has the oar in the gluconeogenesis boat and when? World J Diabetes. 2023;14(7):1049–56. 10.4239/wjd.v14.i7.1049. 37547592 10.4239/wjd.v14.i7.1049 PMC10401452

[68] Zhao Q, Han B, Wang L, Wu J, Wang S, Ren Z, et al. AKR1B1-dependent fructose metabolism enhances malignancy of cancer cells. Cell Death Differ. 2024;31(12):1611–24. 10.1038/s41418-024-01393-4. 39406918 10.1038/s41418-024-01393-4 PMC11618507

[69] Andres-Hernando A, Orlicky DJ, Kuwabara M, Cicerchi C, Pedler M, Petrash MJ, et al. Endogenous Fructose Production and Metabolism Drive Metabolic Dysregulation and Liver Disease in Mice with Hereditary Fructose Intolerance. Nutrients. 2023;15(20). 10.3390/nu15204376. 10.3390/nu15204376 PMC10609559 37892451

[70] Medeiros HCD, Lunt SY. The liver converts fructose into lipids to fuel tumours. Nature. 2024;636(8043):580–1. 10.1038/d41586-024-03653-2. 39633121 10.1038/d41586-024-03653-2 PMC11787852

[71] Shapiro A, Mu W, Roncal C, Cheng KY, Johnson RJ, Scarpace PJ. Fructose-induced leptin resistance exacerbates weight gain in response to subsequent high-fat feeding. Am J Physiol Regul Integr Comp Physiol. 2008;295(5):R1370-5. 10.1152/ajpregu.00195.2008. 18703413 10.1152/ajpregu.00195.2008 PMC2584858

[72] Teff KL, Elliott SS, Tschöp M, Kieffer TJ, Rader D, Heiman M, et al. Dietary fructose reduces circulating insulin and leptin, attenuates postprandial suppression of ghrelin, and increases triglycerides in women. J Clin Endocrinol Metab. 2004;89(6):2963–72. 10.1210/jc.2003-031855. 15181085 10.1210/jc.2003-031855

[73] DiNicolantonio JJ, Lucan SC, O’Keefe JH. The Evidence for Saturated Fat and for Sugar Related to Coronary Heart Disease. Prog Cardiovasc Dis. 2016;58(5):464–72. 10.1016/j.pcad.2015.11.006. 26586275 10.1016/j.pcad.2015.11.006 PMC4856550

[74] Hothersall EJ, Livingstone SJ, Looker HC, Ahmed SF, Cleland S, Leese GP, et al. Contemporary risk of hip fracture in type 1 and type 2 diabetes: a national registry study from Scotland. J Bone Miner Res. 2014;29(5):1054–60. 10.1002/jbmr.2118. 24155126 10.1002/jbmr.2118 PMC4255308

[75] Sellmeyer DE, Civitelli R, Hofbauer LC, Khosla S, Lecka-Czernik B, Schwartz AV. Skeletal Metabolism, Fracture Risk, and Fracture Outcomes in Type 1 and Type 2 Diabetes. Diabetes. 2016;65(7):1757–66. 10.2337/db16-0063. 27329951 10.2337/db16-0063 PMC4915586

[76] Cipriani C, Colangelo L, Santori R, Renella M, Mastrantonio M, Minisola S, et al. The Interplay Between Bone and Glucose Metabolism. Front Endocrinol (Lausanne). 2020;11:122. 10.3389/fendo.2020.00122. 32265831 10.3389/fendo.2020.00122 PMC7105593

[77] Keats EC, Dominguez JM 2nd, Grant MB, Khan ZA. Switch from canonical to noncanonical Wnt signaling mediates high glucose-induced adipogenesis. Stem Cells. 2014;32(6):1649–60. 10.1002/stem.1659. 24496952 10.1002/stem.1659 PMC4037346

[78] Ouyang X, Cirillo P, Sautin Y, McCall S, Bruchette JL, Diehl AM, et al. Fructose consumption as a risk factor for non-alcoholic fatty liver disease. J Hepatol. 2008;48(6):993–9. 10.1016/j.jhep.2008.02.011. 18395287 10.1016/j.jhep.2008.02.011 PMC2423467

[79] Stanhope KL, Schwarz JM, Keim NL, Griffen SC, Bremer AA, Graham JL, et al. Consuming fructose-sweetened, not glucose-sweetened, beverages increases visceral adiposity and lipids and decreases insulin sensitivity in overweight/obese humans. J Clin Invest. 2009;119(5):1322–34. 10.1172/jci37385. 19381015 10.1172/JCI37385 PMC2673878

[80] Galderisi A, Giannini C, Van Name M, Caprio S. Fructose Consumption Contributes to Hyperinsulinemia in Adolescents With Obesity Through a GLP-1-Mediated Mechanism. J Clin Endocrinol Metab. 2019;104(8):3481–90. 10.1210/jc.2019-00161. 30938760 10.1210/jc.2019-00161 PMC6599430

[81] Kyriazis GA, Soundarapandian MM, Tyrberg B. Sweet taste receptor signaling in beta cells mediates fructose-induced potentiation of glucose-stimulated insulin secretion. Proc Natl Acad Sci U S A. 2012;109(8):E524-32. 10.1073/pnas.1115183109. 22315413 10.1073/pnas.1115183109 PMC3286985

[82] Meeprom A, Sompong W, Suwannaphet W, Yibchok-anun S, Adisakwattana S. Grape seed extract supplementation prevents high-fructose diet-induced insulin resistance in rats by improving insulin and adiponectin signalling pathways. Br J Nutr. 2011;106(8):1173–81. 10.1017/s0007114511001589. 21736810 10.1017/S0007114511001589

[83] Li JM, Wang W, Fan CY, Wang MX, Zhang X, Hu QH, et al. Quercetin Preserves β -Cell Mass and Function in Fructose-Induced Hyperinsulinemia through Modulating Pancreatic Akt/FoxO1 Activation. Evid Based Complement Alternat Med. 2013;2013:303902. 10.1155/2013/303902. 23533474 10.1155/2013/303902 PMC3600179

[84] Cabral PD, Hong NJ, Hye Khan MA, Ortiz PA, Beierwaltes WH, Imig JD, et al. Fructose stimulates Na/H exchange activity and sensitizes the proximal tubule to angiotensin II. Hypertension. 2014;63(3):e68-73. 10.1161/hypertensionaha.113.02564. 24379189 10.1161/HYPERTENSIONAHA.113.02564

[85] Johnson RJ, Perez-Pozo SE, Lillo JL, Grases F, Schold JD, Kuwabara M, et al. Fructose increases risk for kidney stones: potential role in metabolic syndrome and heat stress. BMC Nephrol. 2018;19(1):315. 10.1186/s12882-018-1105-0. 30409184 10.1186/s12882-018-1105-0 PMC6225702

[86] Raben A, Vasilaras TH, Møller AC, Astrup A. Sucrose compared with artificial sweeteners: different effects on ad libitum food intake and body weight after 10 wk of supplementation in overweight subjects. Am J Clin Nutr. 2002;76(4):721–9. 10.1093/ajcn/76.4.721. 12324283 10.1093/ajcn/76.4.721

[87] Tordoff MG, Alleva AM. Effect of drinking soda sweetened with aspartame or high-fructose corn syrup on food intake and body weight. Am J Clin Nutr. 1990;51(6):963–9. 10.1093/ajcn/51.6.963. 2349932 10.1093/ajcn/51.6.963

[88] Do MH, Lee E, Oh MJ, Kim Y, Park HY. High-Glucose or -Fructose Diet Cause Changes of the Gut Microbiota and Metabolic Disorders in Mice without Body Weight Change. Nutrients. 2018;10(6). 10.3390/nu10060761. 10.3390/nu10060761 PMC6024874 29899272

[89] Roncal-Jimenez CA, Lanaspa MA, Rivard CJ, Nakagawa T, Sanchez-Lozada LG, Jalal D, et al. Sucrose induces fatty liver and pancreatic inflammation in male breeder rats independent of excess energy intake. Metabolism. 2011;60(9):1259–70. 10.1016/j.metabol.2011.01.008. 21489572 10.1016/j.metabol.2011.01.008 PMC3137694

[90] Deng S, Ge Y, Zhai Z, Liu H, Zhang X, Chen Y, et al. Fructose induces hepatic steatosis in adolescent mice linked to the disorders of lipid metabolism, bile acid metabolism, and autophagy. J Nutr Biochem. 2024;129:109635. 10.1016/j.jnutbio.2024.109635. 38561080 10.1016/j.jnutbio.2024.109635

[91] Sigala DM, Widaman AM, Hieronimus B, Nunez MV, Lee V, Benyam Y, et al. Effects of Consuming Sugar-Sweetened Beverages for 2 Weeks on 24-h Circulating Leptin Profiles, Ad Libitum Food Intake and Body Weight in Young Adults. Nutrients. 2020;12(12). 10.3390/nu12123893. 10.3390/nu12123893 PMC7765993 33352724

[92] Sievenpiper JL, de Souza RJ, Mirrahimi A, Yu ME, Carleton AJ, Beyene J, et al. Effect of fructose on body weight in controlled feeding trials: a systematic review and meta-analysis. Ann Intern Med. 2012;156(4):291–304. 10.7326/0003-4819-156-4-201202210-00007. 22351714 10.7326/0003-4819-156-4-201202210-00007

[93] Johnson RJ, Lanaspa MA, Sanchez-Lozada LG, Tolan D, Nakagawa T, Ishimoto T, et al. The fructose survival hypothesis for obesity. Philos Trans R Soc Lond B Biol Sci. 1885;2023(378):20220230. 10.1098/rstb.2022.0230. 10.1098/rstb.2022.0230 PMC10363705 37482773

[94] Shapiro A, Tümer N, Gao Y, Cheng KY, Scarpace PJ. Prevention and reversal of diet-induced leptin resistance with a sugar-free diet despite high fat content. Br J Nutr. 2011;106(3):390–7. 10.1017/s000711451100033x. 21418711 10.1017/S000711451100033X

[95] Taylor SR, Ramsamooj S, Liang RJ, Katti A, Pozovskiy R, Vasan N, et al. Dietary fructose improves intestinal cell survival and nutrient absorption. Nature. 2021;597(7875):263–7. 10.1038/s41586-021-03827-2. 34408323 10.1038/s41586-021-03827-2 PMC8686685

[96] Lim JS, Mietus-Snyder M, Valente A, Schwarz JM, Lustig RH. The role of fructose in the pathogenesis of NAFLD and the metabolic syndrome. Nat Rev Gastroenterol Hepatol. 2010;7(5):251–64. 10.1038/nrgastro.2010.41. 20368739 10.1038/nrgastro.2010.41

[97] Jensen T, Abdelmalek MF, Sullivan S, Nadeau KJ, Green M, Roncal C, et al. Fructose and sugar: A major mediator of non-alcoholic fatty liver disease. J Hepatol. 2018;68(5):1063–75. 10.1016/j.jhep.2018.01.019. 29408694 10.1016/j.jhep.2018.01.019 PMC5893377

[98] Softic S, Cohen DE, Kahn CR. Role of Dietary Fructose and Hepatic De Novo Lipogenesis in Fatty Liver Disease. Dig Dis Sci. 2016;61(5):1282–93. 10.1007/s10620-016-4054-0. 26856717 10.1007/s10620-016-4054-0 PMC4838515

[99] Geidl-Flueck B, Hochuli M, Németh Á, Eberl A, Derron N, Köfeler HC, et al. Fructose- and sucrose- but not glucose-sweetened beverages promote hepatic de novo lipogenesis: A randomized controlled trial. J Hepatol. 2021;75(1):46–54. 10.1016/j.jhep.2021.02.027. 33684506 10.1016/j.jhep.2021.02.027

[100] Lê KA, Ith M, Kreis R, Faeh D, Bortolotti M, Tran C, et al. Fructose overconsumption causes dyslipidemia and ectopic lipid deposition in healthy subjects with and without a family history of type 2 diabetes. Am J Clin Nutr. 2009;89(6):1760–5. 10.3945/ajcn.2008.27336. 19403641 10.3945/ajcn.2008.27336

[101] McGarry JD. Banting lecture 2001: dysregulation of fatty acid metabolism in the etiology of type 2 diabetes. Diabetes. 2002;51(1):7–18. 10.2337/diabetes.51.1.7. 11756317 10.2337/diabetes.51.1.7

[102] Herman MA, Samuel VT. The Sweet Path to Metabolic Demise: Fructose and Lipid Synthesis. Trends Endocrinol Metab. 2016;27(10):719–30. 10.1016/j.tem.2016.06.005. 27387598 10.1016/j.tem.2016.06.005 PMC5035631

[103] Orozco JM, Krawczyk PA, Scaria SM, Cangelosi AL, Chan SH, Kunchok T, et al. Dihydroxyacetone phosphate signals glucose availability to mTORC1. Nat Metab. 2020;2(9):893–901. 10.1038/s42255-020-0250-5. 32719541 10.1038/s42255-020-0250-5 PMC7995735

[104] Chhabra R, O’Keefe JH, Patil H, O’Keefe E, Thompson RC, Ansari S, et al. Association of coronary artery calcification with hepatic steatosis in asymptomatic individuals. Mayo Clin Proc. 2013;88(11):1259–65. 10.1016/j.mayocp.2013.06.025. 24138963 10.1016/j.mayocp.2013.06.025

[105] Schwarz JM, Noworolski SM, Wen MJ, Dyachenko A, Prior JL, Weinberg ME, et al. Effect of a High-Fructose Weight-Maintaining Diet on Lipogenesis and Liver Fat. J Clin Endocrinol Metab. 2015;100(6):2434–42. 10.1210/jc.2014-3678. 25825943 10.1210/jc.2014-3678 PMC4454806

[106] Faeh D, Minehira K, Schwarz JM, Periasamy R, Park S, Tappy L. Effect of fructose overfeeding and fish oil administration on hepatic de novo lipogenesis and insulin sensitivity in healthy men. Diabetes. 2005;54(7):1907–13. 10.2337/diabetes.54.7.1907. 15983189 10.2337/diabetes.54.7.1907

[107] Stanhope KL. More pieces of the fructose puzzle. J Intern Med. 2017;282(2):202–4. 10.1111/joim.12644. 28646607 10.1111/joim.12644

[108] Taskinen MR, Söderlund S, Bogl LH, Hakkarainen A, Matikainen N, Pietiläinen KH, et al. Adverse effects of fructose on cardiometabolic risk factors and hepatic lipid metabolism in subjects with abdominal obesity. J Intern Med. 2017;282(2):187–201. 10.1111/joim.12632. 28548281 10.1111/joim.12632

[109] Schwarz JM, Noworolski SM, Erkin-Cakmak A, Korn NJ, Wen MJ, Tai VW, et al. Effects of Dietary Fructose Restriction on Liver Fat, De Novo Lipogenesis, and Insulin Kinetics in Children With Obesity. Gastroenterology. 2017;153(3):743–52. 10.1053/j.gastro.2017.05.043. 28579536 10.1053/j.gastro.2017.05.043 PMC5813289

[110] Zhang AMY, Wellberg EA, Kopp JL, Johnson JD. Hyperinsulinemia in Obesity, Inflammation, and Cancer. Diabetes Metab J. 2021;45(3):285–311. 10.4093/dmj.2020.0250. 33775061 10.4093/dmj.2020.0250 PMC8164941

[111] Bojsen-Møller KN, Lundsgaard AM, Madsbad S, Kiens B, Holst JJ. Hepatic Insulin Clearance in Regulation of Systemic Insulin Concentrations-Role of Carbohydrate and Energy Availability. Diabetes. 2018;67(11):2129–36. 10.2337/db18-0539. 30348819 10.2337/db18-0539

[112] Najjar SM, Perdomo G. Hepatic Insulin Clearance: Mechanism and Physiology. Physiology (Bethesda). 2019;34(3):198–215. 10.1152/physiol.00048.2018. 30968756 10.1152/physiol.00048.2018 PMC6734066

[113] Tokarz VL, MacDonald PE, Klip A. The cell biology of systemic insulin function. J Cell Biol. 2018;217(7):2273–89. 10.1083/jcb.201802095. 29622564 10.1083/jcb.201802095 PMC6028526

[114] Ludwig DS, Ebbeling CB. The Carbohydrate-Insulin Model of Obesity: Beyond “Calories In, Calories Out.” JAMA Intern Med. 2018;178(8):1098–103. 10.1001/jamainternmed.2018.2933. 29971406 10.1001/jamainternmed.2018.2933 PMC6082688

[115] Ludwig DS, Ebbeling CB, Bikman BT, Johnson JD. Testing the carbohydrate-insulin model in mice: The importance of distinguishing primary hyperinsulinemia from insulin resistance and metabolic dysfunction. Mol Metab. 2020;35:100960. 10.1016/j.molmet.2020.02.003. 32199816 10.1016/j.molmet.2020.02.003 PMC7191247

[116] Lebovitz HE. Insulin resistance: definition and consequences. Exp Clin Endocrinol Diabetes. 2001;109(Suppl 2):S135-48. 10.1055/s-2001-18576. 11460565 10.1055/s-2001-18576

[117] Hill MA, Yang Y, Zhang L, Sun Z, Jia G, Parrish AR, et al. Insulin resistance, cardiovascular stiffening and cardiovascular disease. Metabolism. 2021;119:154766. 10.1016/j.metabol.2021.154766. 33766485 10.1016/j.metabol.2021.154766

[118] Abdul-Ghani M, DeFronzo RA. Insulin Resistance and Hyperinsulinemia: the Egg and the Chicken. J Clin Endocrinol Metab. 2021;106(4):e1897–9. 10.1210/clinem/dgaa364. 33522574 10.1210/clinem/dgaa364 PMC7993580

[119] Tran LT, Yuen VG, McNeill JH. The fructose-fed rat: a review on the mechanisms of fructose-induced insulin resistance and hypertension. Mol Cell Biochem. 2009;332(1–2):145–59. 10.1007/s11010-009-0184-4. 19536638 10.1007/s11010-009-0184-4

[120] DeFronzo RA. Pathogenesis of type 2 diabetes mellitus. Med Clin North Am. 2004;88(4):787–835, x. 10.1016/j.mcna.2004.04.013. 15308380 10.1016/j.mcna.2004.04.013

[121] DeFronzo RA. Insulin resistance, hyperinsulinemia, and coronary artery disease: a complex metabolic web. J Cardiovasc Pharmacol. 1992;20(Suppl 11):S1-16. 10.1097/00005344-199200111-00002. 1284137 10.1097/00005344-199200111-00002

[122] Reiser S, Michaelis OEt, Cataland S, O’Dorisio TM. Effect of isocaloric exchange of dietary starch and sucrose in humans on the gastric inhibitory polypeptide response to a sucrose load. Am J Clin Nutr. 1980;33(9):1907–11. 10.1093/ajcn/33.9.1907. 6998274 10.1093/ajcn/33.9.1907

[123] Hallfrisch J, Ellwood KC, Michaelis OEt, Reiser S, O’Dorisio TM, Prather ES. Effects of dietary fructose on plasma glucose and hormone responses in normal and hyperinsulinemic men. J Nutr. 1983;113(9):1819–26. 10.1093/jn/113.9.1819. 6350544 10.1093/jn/113.9.1819

[124] Hwang IS, Ho H, Hoffman BB, Reaven GM. Fructose-induced insulin resistance and hypertension in rats. Hypertension. 1987;10(5):512–6. 10.1161/01.hyp.10.5.512. 3311990 10.1161/01.hyp.10.5.512

[125] Pagliassotti MJ, Prach PA. Quantity of sucrose alters the tissue pattern and time course of insulin resistance in young rats. Am J Physiol. 1995;269(3 Pt 2):R641-6. 10.1152/ajpregu.1995.269.3.R641. 7573567 10.1152/ajpregu.1995.269.3.R641

[126] Anuradha CV, Balakrishnan SD. Taurine attenuates hypertension and improves insulin sensitivity in the fructose-fed rat, an animal model of insulin resistance. Can J Physiol Pharmacol. 1999;77(10):749–54. 10588478

[127] Dunnigan MG, Ford JA. The insulin response to intravenous fructose in relation to blood glucose levels. J Clin Endocrinol Metab. 1975;40(4):629–35. 10.1210/jcem-40-4-629. 1127073 10.1210/jcem-40-4-629

[128] Kuhre RE, Gribble FM, Hartmann B, Reimann F, Windeløv JA, Rehfeld JF, et al. Fructose stimulates GLP-1 but not GIP secretion in mice, rats, and humans. Am J Physiol Gastrointest Liver Physiol. 2014;306(7):G622-30. 10.1152/ajpgi.00372.2013. 24525020 10.1152/ajpgi.00372.2013 PMC3962593

[129] Reimann F, Habib AM, Tolhurst G, Parker HE, Rogers GJ, Gribble FM. Glucose sensing in L cells: a primary cell study. Cell Metab. 2008;8(6):532–9. 10.1016/j.cmet.2008.11.002. 19041768 10.1016/j.cmet.2008.11.002 PMC2697331

[130] Morioka T, Asilmaz E, Hu J, Dishinger JF, Kurpad AJ, Elias CF, et al. Disruption of leptin receptor expression in the pancreas directly affects beta cell growth and function in mice. J Clin Invest. 2007;117(10):2860–8. 10.1172/jci30910. 17909627 10.1172/JCI30910 PMC1994606

[131] Bernal-Mizrachi E, Wen W, Stahlhut S, Welling CM, Permutt MA. Islet beta cell expression of constitutively active Akt1/PKB alpha induces striking hypertrophy, hyperplasia, and hyperinsulinemia. J Clin Invest. 2001;108(11):1631–8. 10.1172/jci13785. 11733558 10.1172/JCI13785 PMC200992

[132] Tuttle RL, Gill NS, Pugh W, Lee JP, Koeberlein B, Furth EE, et al. Regulation of pancreatic beta-cell growth and survival by the serine/threonine protein kinase Akt1/PKBalpha. Nat Med. 2001;7(10):1133–7. 10.1038/nm1001-1133. 11590437 10.1038/nm1001-1133

[133] Tsai HY, Wu LY, Hwang LS. Effect of a proanthocyanidin-rich extract from longan flower on markers of metabolic syndrome in fructose-fed rats. J Agric Food Chem. 2008;56(22):11018–24. 10.1021/jf801966y. 18973337 10.1021/jf801966y

[134] Cao H, Hininger-Favier I, Kelly MA, Benaraba R, Dawson HD, Coves S, et al. Green tea polyphenol extract regulates the expression of genes involved in glucose uptake and insulin signaling in rats fed a high fructose diet. J Agric Food Chem. 2007;55(15):6372–8. 10.1021/jf070695o. 17616136 10.1021/jf070695o

[135] Stefan N, Vozarova B, Funahashi T, Matsuzawa Y, Weyer C, Lindsay RS, et al. Plasma adiponectin concentration is associated with skeletal muscle insulin receptor tyrosine phosphorylation, and low plasma concentration precedes a decrease in whole-body insulin sensitivity in humans. Diabetes. 2002;51(6):1884–8. 10.2337/diabetes.51.6.1884. 12031977 10.2337/diabetes.51.6.1884

[136] Lindsay RS, Funahashi T, Hanson RL, Matsuzawa Y, Tanaka S, Tataranni PA, et al. Adiponectin and development of type 2 diabetes in the Pima Indian population. Lancet. 2002;360(9326):57–8. 10.1016/s0140-6736(02)09335-2. 12114044 10.1016/S0140-6736(02)09335-2

[137] Bonnard C, Durand A, Vidal H, Rieusset J. Changes in adiponectin, its receptors and AMPK activity in tissues of diet-induced diabetic mice. Diabetes Metab. 2008;34(1):52–61. 10.1016/j.diabet.2007.09.006. 18222103 10.1016/j.diabet.2007.09.006

[138] Fathallah-Shaykh SA, Cramer MT. Uric acid and the kidney. Pediatr Nephrol. 2014;29(6):999–1008. 10.1007/s00467-013-2549-x. 23824181 10.1007/s00467-013-2549-x

[139] Glantzounis GK, Tsimoyiannis EC, Kappas AM, Galaris DA. Uric acid and oxidative stress. Curr Pharm Des. 2005;11(32):4145–51. 10.2174/138161205774913255. 16375736 10.2174/138161205774913255

[140] Tao L, Li D, Li Y, Shi X, Wang J, Rao C, et al. Designing a mutant Candida uricase with improved polymerization state and enzymatic activity. Protein Eng Des Sel. 2017;30(11):753–9. 10.1093/protein/gzx056. 29161434 10.1093/protein/gzx056

[141] Maxwell SR, Thomason H, Sandler D, Leguen C, Baxter MA, Thorpe GH, et al. Antioxidant status in patients with uncomplicated insulin-dependent and non-insulin-dependent diabetes mellitus. Eur J Clin Invest. 1997;27(6):484–90. 10.1046/j.1365-2362.1997.1390687.x. 9229228 10.1046/j.1365-2362.1997.1390687.x

[142] Ames BN, Cathcart R, Schwiers E, Hochstein P. Uric acid provides an antioxidant defense in humans against oxidant- and radical-caused aging and cancer: a hypothesis. Proc Natl Acad Sci U S A. 1981;78(11):6858–62. 10.1073/pnas.78.11.6858. 6947260 10.1073/pnas.78.11.6858 PMC349151

[143] Becker BF. Towards the physiological function of uric acid. Free Radic Biol Med. 1993;14(6):615–31. 10.1016/0891-5849(93)90143-i. 8325534 10.1016/0891-5849(93)90143-i

[144] Itahana Y, Han R, Barbier S, Lei Z, Rozen S, Itahana K. The uric acid transporter SLC2A9 is a direct target gene of the tumor suppressor p53 contributing to antioxidant defense. Oncogene. 2015;34(14):1799–810. 10.1038/onc.2014.119. 24858040 10.1038/onc.2014.119

[145] Wang Q, Wen X, Kong J. Recent Progress on Uric Acid Detection: A Review. Crit Rev Anal Chem. 2020;50(4):359–75. 10.1080/10408347.2019.1637711. 31296022 10.1080/10408347.2019.1637711

[146] Perez-Ruiz F, Dalbeth N, Bardin T. A review of uric acid, crystal deposition disease, and gout. Adv Ther. 2015;32(1):31–41. 10.1007/s12325-014-0175-z. 25533440 10.1007/s12325-014-0175-z PMC4311063

[147] Kuo CF, Grainge MJ, Zhang W, Doherty M. Global epidemiology of gout: prevalence, incidence and risk factors. Nat Rev Rheumatol. 2015;11(11):649–62. 10.1038/nrrheum.2015.91. 26150127 10.1038/nrrheum.2015.91

[148] Dalbeth N, Gosling AL, Gaffo A, Abhishek A. Gout Lancet. 2021;397(10287):1843–55. 10.1016/s0140-6736(21)00569-9. 33798500 10.1016/S0140-6736(21)00569-9

[149] Lubawy M, Formanowicz D. High-Fructose Diet-Induced Hyperuricemia Accompanying Metabolic Syndrome-Mechanisms and Dietary Therapy Proposals. Int J Environ Res Public Health. 2023;20(4). 10.3390/ijerph20043596. 10.3390/ijerph20043596 PMC9960726 36834291

[150] Jamnik J, Rehman S, Blanco Mejia S, de Souza RJ, Khan TA, Leiter LA, et al. Fructose intake and risk of gout and hyperuricemia: a systematic review and meta-analysis of prospective cohort studies. BMJ Open. 2016;6(10):e013191. 10.1136/bmjopen-2016-013191. 27697882 10.1136/bmjopen-2016-013191 PMC5073537

[151] Reiser S, Powell AS, Scholfield DJ, Panda P, Ellwood KC, Canary JJ. Blood lipids, lipoproteins, apoproteins, and uric acid in men fed diets containing fructose or high-amylose cornstarch. Am J Clin Nutr. 1989;49(5):832–9. 10.1093/ajcn/49.5.832. 2497634 10.1093/ajcn/49.5.832

[152] Hallfrisch J, Ellwood K, Michaelis OEt, Reiser S, Prather ES. Plasma fructose uric acid and inorganic phosphorus responses of hyperinsulinemic men fed fructose. J Am Coll Nutr. 1986;5(1):61–8. 10.1080/07315724.1986.10720113. 3517112 10.1080/07315724.1986.10720113

[153] Baharuddin B. The Impact of Fructose Consumption on Human Health: Effects on Obesity, Hyperglycemia, Diabetes, Uric Acid, and Oxidative Stress With a Focus on the Liver. Cureus. 2024;16(9):e70095. 10.7759/cureus.70095. 39355469 10.7759/cureus.70095 PMC11444807

[154] Johnson RJ, Nakagawa T, Sanchez-Lozada LG, Shafiu M, Sundaram S, Le M, et al. Sugar, uric acid, and the etiology of diabetes and obesity. Diabetes. 2013;62(10):3307–15. 10.2337/db12-1814. 24065788 10.2337/db12-1814 PMC3781481

[155] Kaneko C, Ogura J, Sasaki S, Okamoto K, Kobayashi M, Kuwayama K, et al. Fructose suppresses uric acid excretion to the intestinal lumen as a result of the induction of oxidative stress by NADPH oxidase activation. Biochim Biophys Acta Gen Subj. 2017;1861(3):559–66. 10.1016/j.bbagen.2016.11.042. 27913188 10.1016/j.bbagen.2016.11.042

[156] Caliceti C, Calabria D, Roda A, Cicero AFG. Fructose Intake, Serum Uric Acid, and Cardiometabolic Disorders: A Critical Review. Nutrients. 2017;9(4). 10.3390/nu9040395. 10.3390/nu9040395 PMC5409734 28420204

[157] Song 宋志林 Z, Roncal-Jimenez CA, Lanaspa-Garcia MA, Oppelt SA, Kuwabara M, Jensen T, et al. Role of fructose and fructokinase in acute dehydration-induced vasopressin gene expression and secretion in mice. J Neurophysiol. 2017;117(2):646–54. 10.1152/jn.00781.2016. 10.1152/jn.00781.2016 PMC5288484 27852737

[158] Zhang C, Li L, Zhang Y, Zeng C. Recent advances in fructose intake and risk of hyperuricemia. Biomed Pharmacother. 2020;131:110795. 10.1016/j.biopha.2020.110795. 33152951 10.1016/j.biopha.2020.110795

[159] Zhang DM, Jiao RQ, Kong LD. High Dietary Fructose: Direct or Indirect Dangerous Factors Disturbing Tissue and Organ Functions. Nutrients. 2017;9(4). 10.3390/nu9040335. 10.3390/nu9040335 PMC5409674 28353649

[160] Komnenov D, Levanovich PE, Rossi NF. Hypertension Associated with Fructose and High Salt: Renal and Sympathetic Mechanisms. Nutrients. 2019;11(3). 10.3390/nu11030569. 10.3390/nu11030569 PMC6472002 30866441

[161] Carretero OA, Oparil S. Essential hypertension. Part I: definition and etiology Circulation. 2000;101(3):329–35. 10.1161/01.cir.101.3.329. 10645931 10.1161/01.cir.101.3.329

[162] Sliwa K, Stewart S, Gersh BJ. Hypertension: a global perspective. Circulation. 2011;123(24):2892–6. 10.1161/circulationaha.110.992362. 21690504 10.1161/CIRCULATIONAHA.110.992362

[163] Russo E, Leoncini G, Esposito P, Garibotto G, Pontremoli R, Viazzi F. Fructose and Uric Acid: Major Mediators of Cardiovascular Disease Risk Starting at Pediatric Age. Int J Mol Sci. 2020;21(12). 10.3390/ijms21124479. 10.3390/ijms21124479 PMC7352635 32599713

[164] Bray GA. Fructose and risk of cardiometabolic disease. Curr Atheroscler Rep. 2012;14(6):570–8. 10.1007/s11883-012-0276-6. 22949106 10.1007/s11883-012-0276-6 PMC8407053

[165] Shi YN, Liu YJ, Xie Z, Zhang WJ. Fructose and metabolic diseases: too much to be good. Chin Med J (Engl). 2021;134(11):1276–85. 10.1097/cm9.0000000000001545. 34010200 10.1097/CM9.0000000000001545 PMC8183764

[166] Reaven GM, Ho H, Hoffman BB. Attenuation of fructose-induced hypertension in rats by exercise training. Hypertension. 1988;12(2):129–32. 10.1161/01.hyp.12.2.129. 3410522 10.1161/01.hyp.12.2.129

[167] Brown CM, Dulloo AG, Yepuri G, Montani JP. Fructose ingestion acutely elevates blood pressure in healthy young humans. Am J Physiol Regul Integr Comp Physiol. 2008;294(3):R730-7. 10.1152/ajpregu.00680.2007. 18199590 10.1152/ajpregu.00680.2007

[168] Tappy L, Randin JP, Felber JP, Chiolero R, Simonson DC, Jequier E, et al. Comparison of thermogenic effect of fructose and glucose in normal humans. Am J Physiol. 1986;250(6 Pt 1):E718-24. 10.1152/ajpendo.1986.250.6.E718. 3521319 10.1152/ajpendo.1986.250.6.E718

[169] Hsieh PS, Tai YH, Loh CH, Shih KC, Cheng WT, Chu CH. Functional interaction of AT1 and AT2 receptors in fructose-induced insulin resistance and hypertension in rats. Metabolism. 2005;54(2):157–64. 10.1016/j.metabol.2004.07.016. 15690308 10.1016/j.metabol.2004.07.016

[170] Juan CC, Fang VS, Hsu YP, Huang YJ, Hsia DB, Yu PC, et al. Overexpression of vascular endothelin-1 and endothelin-A receptors in a fructose-induced hypertensive rat model. J Hypertens. 1998;16(12 Pt 1):1775–82. 10.1097/00004872-199816120-00010. 9869011 10.1097/00004872-199816120-00010

[171] Gray C, Gardiner SM, Elmes M, Gardner DS. Excess maternal salt or fructose intake programmes sex-specific, stress- and fructose-sensitive hypertension in the offspring. Br J Nutr. 2016;115(4):594–604. 10.1017/s0007114515004936. 26653028 10.1017/S0007114515004936

[172] Li N, Zhang S, Li W, Wang L, Liu H, Li W, et al. Prevalence of hyperuricemia and its related risk factors among preschool children from China. Sci Rep. 2017;7(1):9448. 10.1038/s41598-017-10120-8. 28842671 10.1038/s41598-017-10120-8 PMC5573349

[173] Viazzi F, Antolini L, Giussani M, Brambilla P, Galbiati S, Mastriani S, et al. Serum uric acid and blood pressure in children at cardiovascular risk. Pediatrics. 2013;132(1):e93-9. 10.1542/peds.2013-0047. 23776119 10.1542/peds.2013-0047

[174] Johnson RJ, Segal MS, Sautin Y, Nakagawa T, Feig DI, Kang DH, et al. Potential role of sugar (fructose) in the epidemic of hypertension, obesity and the metabolic syndrome, diabetes, kidney disease, and cardiovascular disease. Am J Clin Nutr. 2007;86(4):899–906. 10.1093/ajcn/86.4.899. 17921363 10.1093/ajcn/86.4.899

[175] Choi YJ, Yoon Y, Lee KY, Hien TT, Kang KW, Kim KC, et al. Uric acid induces endothelial dysfunction by vascular insulin resistance associated with the impairment of nitric oxide synthesis. Faseb j. 2014;28(7):3197–204. 10.1096/fj.13-247148. 24652948 10.1096/fj.13-247148

[176] Johnson RJ, Segal MS, Srinivas T, Ejaz A, Mu W, Roncal C, et al. Essential hypertension, progressive renal disease, and uric acid: a pathogenetic link? J Am Soc Nephrol. 2005;16(7):1909–19. 10.1681/asn.2005010063. 15843466 10.1681/ASN.2005010063

[177] Grayson PC, Kim SY, LaValley M, Choi HK. Hyperuricemia and incident hypertension: a systematic review and meta-analysis. Arthritis Care Res (Hoboken). 2011;63(1):102–10. 10.1002/acr.20344. 20824805 10.1002/acr.20344 PMC3016454

[178] Cirillo P, Gersch MS, Mu W, Scherer PM, Kim KM, Gesualdo L, et al. Ketohexokinase-dependent metabolism of fructose induces proinflammatory mediators in proximal tubular cells. J Am Soc Nephrol. 2009;20(3):545–53. 10.1681/asn.2008060576. 19158351 10.1681/ASN.2008060576 PMC2653686

[179] Sánchez-Lozada LG, Tapia E, Bautista-García P, Soto V, Avila-Casado C, Vega-Campos IP, et al. Effects of febuxostat on metabolic and renal alterations in rats with fructose-induced metabolic syndrome. Am J Physiol Renal Physiol. 2008;294(4):F710-8. 10.1152/ajprenal.00454.2007. 18216151 10.1152/ajprenal.00454.2007

[180] Gordish KL, Kassem KM, Ortiz PA, Beierwaltes WH. Moderate (20%) fructose-enriched diet stimulates salt-sensitive hypertension with increased salt retention and decreased renal nitric oxide. Physiol Rep. 2017;5(7). 10.14814/phy2.13162. 10.14814/phy2.13162 PMC5392503 28408634

[181] Zeng W, Pirzgalska RM, Pereira MM, Kubasova N, Barateiro A, Seixas E, et al. Sympathetic neuro-adipose connections mediate leptin-driven lipolysis. Cell. 2015;163(1):84–94. 10.1016/j.cell.2015.08.055. 26406372 10.1016/j.cell.2015.08.055 PMC7617198

[182] Soleimani M, Alborzi P. The role of salt in the pathogenesis of fructose-induced hypertension. Int J Nephrol. 2011;2011:392708. 10.4061/2011/392708. 21789281 10.4061/2011/392708 PMC3140039

[183] Lancaster KJ. Current Intake and Demographic Disparities in the Association of Fructose-Rich Foods and Metabolic Syndrome. JAMA Netw Open. 2020;3(7):e2010224. 10.1001/jamanetworkopen.2020.10224. 32644135 10.1001/jamanetworkopen.2020.10224

[184] Miller A, Adeli K. Dietary fructose and the metabolic syndrome. Curr Opin Gastroenterol. 2008;24(2):204–9. 10.1097/MOG.0b013e3282f3f4c4. 18301272 10.1097/MOG.0b013e3282f3f4c4

[185] Wang X, Xu Z, Chang R, Zeng C, Zhao Y. High-Fructose Diet Induces Cardiac Dysfunction via Macrophage Recruitment in Adult Mice. J Cardiovasc Pharmacol Ther. 2023;28:10742484231162248. 10.1177/10742484231162249. 36995038 10.1177/10742484231162249

[186] Vasiljević A, Bursać B, Djordjevic A, Milutinović DV, Nikolić M, Matić G, et al. Hepatic inflammation induced by high-fructose diet is associated with altered 11βHSD1 expression in the liver of Wistar rats. Eur J Nutr. 2014;53(6):1393–402. 10.1007/s00394-013-0641-4. 24389792 10.1007/s00394-013-0641-4

[187] Sellmann C, Priebs J, Landmann M, Degen C, Engstler AJ, Jin CJ, et al. Diets rich in fructose, fat or fructose and fat alter intestinal barrier function and lead to the development of nonalcoholic fatty liver disease over time. J Nutr Biochem. 2015;26(11):1183–92. 10.1016/j.jnutbio.2015.05.011. 26168700 10.1016/j.jnutbio.2015.05.011

[188] Porto ML, Lírio LM, Dias AT, Batista AT, Campagnaro BP, Mill JG, et al. Increased oxidative stress and apoptosis in peripheral blood mononuclear cells of fructose-fed rats. Toxicol In Vitro. 2015;29(8):1977–81. 10.1016/j.tiv.2015.08.006. 26279319 10.1016/j.tiv.2015.08.006

[189] Hsu TM, Konanur VR, Taing L, Usui R, Kayser BD, Goran MI, et al. Effects of sucrose and high fructose corn syrup consumption on spatial memory function and hippocampal neuroinflammation in adolescent rats. Hippocampus. 2015;25(2):227–39. 10.1002/hipo.22368. 25242636 10.1002/hipo.22368

[190] Tan R, Dong H, Chen Z, Jin M, Yin J, Li H, et al. Intestinal Microbiota Mediates High-Fructose and High-Fat Diets to Induce Chronic Intestinal Inflammation. Front Cell Infect Microbiol. 2021;11:654074. 10.3389/fcimb.2021.654074. 34222037 10.3389/fcimb.2021.654074 PMC8242949

[191] Seki K, Kitade M, Nishimura N, Kaji K, Asada K, Namisaki T, et al. Oral administration of fructose exacerbates liver fibrosis and hepatocarcinogenesis via increased intestinal permeability in a rat steatohepatitis model. Oncotarget. 2018;9(47):28638–51. 10.18632/oncotarget.25587. 10.18632/oncotarget.25587 PMC6033350 29983886

[192] Li JM, Yu R, Zhang LP, Wen SY, Wang SJ, Zhang XY, et al. Dietary fructose-induced gut dysbiosis promotes mouse hippocampal neuroinflammation: a benefit of short-chain fatty acids. Microbiome. 2019;7(1):98. 10.1186/s40168-019-0713-7. 31255176 10.1186/s40168-019-0713-7 PMC6599330

[193] Xu MX, Yu R, Shao LF, Zhang YX, Ge CX, Liu XM, et al. Up-regulated fractalkine (FKN) and its receptor CX3CR1 are involved in fructose-induced neuroinflammation: Suppression by curcumin. Brain Behav Immun. 2016;58:69–81. 10.1016/j.bbi.2016.01.001. 26765996 10.1016/j.bbi.2016.01.001

[194] Chen L, Lan Z, Lin Q, Mi X, He Y, Wei L, et al. Polydatin ameliorates renal injury by attenuating oxidative stress-related inflammatory responses in fructose-induced urate nephropathic mice. Food Chem Toxicol. 2013;52:28–35. 10.1016/j.fct.2012.10.037. 23137955 10.1016/j.fct.2012.10.037

[195] Kovačević S, Nestorov J, Matić G, Elaković I. Fructose-enriched diet induces inflammation and reduces antioxidative defense in visceral adipose tissue of young female rats. Eur J Nutr. 2017;56(1):151–60. 10.1007/s00394-015-1065-0. 26433940 10.1007/s00394-015-1065-0

[196] Ren X, Xu J, Xu Y, Wang Q, Huang K, He X. Artemether Attenuates Gut Barrier Dysfunction and Intestinal Flora Imbalance in High-Fat and High-Fructose Diet-Fed Mice. Nutrients. 2023;15(23). 10.3390/nu15234860. 10.3390/nu15234860 PMC10707945 38068719

[197] Jin R, Willment A, Patel SS, Sun X, Song M, Mannery YO, et al. Fructose induced endotoxemia in pediatric nonalcoholic Fatty liver disease. Int J Hepatol. 2014;2014:560620. 10.1155/2014/560620. 25328713 10.1155/2014/560620 PMC4195259

[198] Volynets V, Louis S, Pretz D, Lang L, Ostaff MJ, Wehkamp J, et al. Intestinal Barrier Function and the Gut Microbiome Are Differentially Affected in Mice Fed a Western-Style Diet or Drinking Water Supplemented with Fructose. J Nutr. 2017;147(5):770–80. 10.3945/jn.116.242859. 28356436 10.3945/jn.116.242859

[199] Kavanagh K, Wylie AT, Tucker KL, Hamp TJ, Gharaibeh RZ, Fodor AA, et al. Dietary fructose induces endotoxemia and hepatic injury in calorically controlled primates. Am J Clin Nutr. 2013;98(2):349–57. 10.3945/ajcn.112.057331. 23783298 10.3945/ajcn.112.057331 PMC3712547

[200] Zhou M, Liu X, He J, Xu X, Ju C, Luo S, et al. High-fructose corn syrup aggravates colitis via microbiota dysbiosis-mediated Th17/Treg imbalance. Clin Sci (Lond). 2023;137(20):1619–35. 10.1042/cs20230788. 37818653 10.1042/CS20230788

[201] Bergheim I, Weber S, Vos M, Krämer S, Volynets V, Kaserouni S, et al. Antibiotics protect against fructose-induced hepatic lipid accumulation in mice: role of endotoxin. J Hepatol. 2008;48(6):983–92. 10.1016/j.jhep.2008.01.035. 18395289 10.1016/j.jhep.2008.01.035

[202] Abdelmegeed MA, Moon KH, Chen C, Gonzalez FJ, Song BJ. Role of cytochrome P450 2E1 in protein nitration and ubiquitin-mediated degradation during acetaminophen toxicity. Biochem Pharmacol. 2010;79(1):57–66. 10.1016/j.bcp.2009.07.016. 19660437 10.1016/j.bcp.2009.07.016 PMC2784150

[203] Cho YE, Kim DK, Seo W, Gao B, Yoo SH, Song BJ. Fructose Promotes Leaky Gut, Endotoxemia, and Liver Fibrosis Through Ethanol-Inducible Cytochrome P450–2E1-Mediated Oxidative and Nitrative Stress. Hepatology. 2021;73(6):2180–95. 10.1002/hep.30652. 30959577 10.1002/hep.30652 PMC6783321

[204] Yalçın Buğdaycı A, Akarca Dizakar S, Demirel MA, Ömeroğlu S, Akar F, Uludağ MO. Investigation of the relationship between inflammation and microbiota in the intestinal tissue of female and male rats fed with fructose: Modulatory role of metformin. Daru. 2024; 10.1007/s40199-024-00521-2. 10.1007/s40199-024-00521-2 PMC11554967 38884844

[205] Ajamian M, Steer D, Rosella G, Gibson PR. Serum zonulin as a marker of intestinal mucosal barrier function: May not be what it seems. PLoS One. 2019;14(1):e0210728. 10.1371/journal.pone.0210728. 30640940 10.1371/journal.pone.0210728 PMC6331146

[206] Cheng WL, Li SJ, Lee TI, Lee TW, Chung CC, Kao YH, et al. Sugar Fructose Triggers Gut Dysbiosis and Metabolic Inflammation with Cardiac Arrhythmogenesis. Biomedicines. 2021;9(7). 10.3390/biomedicines9070728. 10.3390/biomedicines9070728 PMC8301417 34201938

[207] Dorrestein PC, Mazmanian SK, Knight R. Finding the missing links among metabolites, microbes, and the host. Immunity. 2014;40(6):824–32. 10.1016/j.immuni.2014.05.015. 24950202 10.1016/j.immuni.2014.05.015 PMC4503329

[208] Miura K, Ishioka M, Iijima K. The Roles of the Gut Microbiota and Toll-like Receptors in Obesity and Nonalcoholic Fatty Liver Disease. J Obes Metab Syndr. 2017;26(2):86–96. 10.7570/jomes.2017.26.2.86. 31089501 10.7570/jomes.2017.26.2.86 PMC6484897

[209] Tie H-M, Jiang W-D, Feng L, Wu P, Liu Y, Kuang S-Y, et al. Dietary nucleotides in the diets of on-growing grass carp (Ctenopharyngodon idella) suppress Aeromonas hydrophila induced intestinal inflammation and enhance intestinal disease-resistance via NF-κB and TOR signaling. Aquaculture. 2021;533:736075. 10.1016/j.aquaculture.2020.736075.

[210] Xiong T, Zheng X, Zhang K, Wu H, Dong Y, Zhou F, et al. Ganluyin ameliorates DSS-induced ulcerative colitis by inhibiting the enteric-origin LPS/TLR4/NF-κB pathway. J Ethnopharmacol. 2022;289:115001. 10.1016/j.jep.2022.115001. 35085745 10.1016/j.jep.2022.115001

[211] Lim SM, Jeong JJ, Woo KH, Han MJ, Kim DH. Lactobacillus sakei OK67 ameliorates high-fat diet-induced blood glucose intolerance and obesity in mice by inhibiting gut microbiota lipopolysaccharide production and inducing colon tight junction protein expression. Nutr Res. 2016;36(4):337–48. 10.1016/j.nutres.2015.12.001. 27001279 10.1016/j.nutres.2015.12.001

[212] Todoric J, Di Caro G, Reibe S, Henstridge DC, Green CR, Vrbanac A, et al. Fructose stimulated de novo lipogenesis is promoted by inflammation. Nat Metab. 2020;2(10):1034–45. 10.1038/s42255-020-0261-2. 32839596 10.1038/s42255-020-0261-2 PMC8018782

[213] Parada Venegas D, De la Fuente MK, Landskron G, González MJ, Quera R, Dijkstra G, et al. Short Chain Fatty Acids (SCFAs)-Mediated Gut Epithelial and Immune Regulation and Its Relevance for Inflammatory Bowel Diseases. Front Immunol. 2019;10:277. 10.3389/fimmu.2019.00277. 30915065 10.3389/fimmu.2019.00277 PMC6421268

[214] Manichanh C, Borruel N, Casellas F, Guarner F. The gut microbiota in IBD. Nature Reviews Gastroenterology & Hepatology. 2012;9(10):599–608. 10.1038/nrgastro.2012.152. 22907164 10.1038/nrgastro.2012.152

[215] Wu W, Sun M, Chen F, Cao AT, Liu H, Zhao Y, et al. Microbiota metabolite short-chain fatty acid acetate promotes intestinal IgA response to microbiota which is mediated by GPR43. Mucosal Immunol. 2017;10(4):946–56. 10.1038/mi.2016.114. 27966553 10.1038/mi.2016.114 PMC5471141

[216] Fagarasan S, Kawamoto S, Kanagawa O, Suzuki K. Adaptive immune regulation in the gut: T cell-dependent and T cell-independent IgA synthesis. Annu Rev Immunol. 2010;28:243–73. 10.1146/annurev-immunol-030409-101314. 20192805 10.1146/annurev-immunol-030409-101314

[217] Cerutti A, Rescigno M. The biology of intestinal immunoglobulin A responses. Immunity. 2008;28(6):740–50. 10.1016/j.immuni.2008.05.001. 18549797 10.1016/j.immuni.2008.05.001 PMC3057455

[218] Chang PV, Hao L, Offermanns S, Medzhitov R. The microbial metabolite butyrate regulates intestinal macrophage function via histone deacetylase inhibition. Proc Natl Acad Sci U S A. 2014;111(6):2247–52. 10.1073/pnas.1322269111. 24390544 10.1073/pnas.1322269111 PMC3926023

[219] Davie JR. Inhibition of histone deacetylase activity by butyrate. J Nutr. 2003;133(7 Suppl):2485s–93s. 10.1093/jn/133.7.2485S. 12840228 10.1093/jn/133.7.2485S

[220] Sun M, Wu W, Chen L, Yang W, Huang X, Ma C, et al. Microbiota-derived short-chain fatty acids promote Th1 cell IL-10 production to maintain intestinal homeostasis. Nat Commun. 2018;9(1):3555. 10.1038/s41467-018-05901-2. 30177845 10.1038/s41467-018-05901-2 PMC6120873

[221] Smith PM, Howitt MR, Panikov N, Michaud M, Gallini CA, Bohlooly YM, et al. The microbial metabolites, short-chain fatty acids, regulate colonic Treg cell homeostasis. Science. 2013;341(6145):569–73. 10.1126/science.1241165. 23828891 10.1126/science.1241165 PMC3807819

[222] Arpaia N, Campbell C, Fan X, Dikiy S, van der Veeken J, deRoos P, et al. Metabolites produced by commensal bacteria promote peripheral regulatory T-cell generation. Nature. 2013;504(7480):451–5. 10.1038/nature12726. 24226773 10.1038/nature12726 PMC3869884

[223] Furusawa Y, Obata Y, Fukuda S, Endo TA, Nakato G, Takahashi D, et al. Commensal microbe-derived butyrate induces the differentiation of colonic regulatory T cells. Nature. 2013;504(7480):446–50. 10.1038/nature12721. 24226770 10.1038/nature12721

[224] Atarashi K, Tanoue T, Oshima K, Suda W, Nagano Y, Nishikawa H, et al. Treg induction by a rationally selected mixture of Clostridia strains from the human microbiota. Nature. 2013;500(7461):232–6. 10.1038/nature12331. 23842501 10.1038/nature12331

[225] Cohen JC, Horton JD, Hobbs HH. Human fatty liver disease: old questions and new insights. Science. 2011;332(6037):1519–23. 10.1126/science.1204265. 21700865 10.1126/science.1204265 PMC3229276

[226] Van Herck MA, Vonghia L, Francque SM. Animal Models of Nonalcoholic Fatty Liver Disease-A Starter's Guide. Nutrients. 2017;9(10) 10.3390/nu9101072. 10.3390/nu9101072 PMC5691689 28953222

[227] Zhong F, Zhou X, Xu J, Gao L. Rodent Models of Nonalcoholic Fatty Liver Disease. Digestion. 2020;101(5):522–35. 10.1159/000501851. 31600750 10.1159/000501851

[228] Starling S. Role of fructose in NASH defined. Nat Rev Endocrinol. 2020;16(11):624. 10.1038/s41574-020-00419-4. 32887949 10.1038/s41574-020-00419-4

[229] Castro MC, Massa ML, Arbeláez LG, Schinella G, Gagliardino JJ, Francini F. Fructose-induced inflammation, insulin resistance and oxidative stress: A liver pathological triad effectively disrupted by lipoic acid. Life Sci. 2015;137:1–6. 10.1016/j.lfs.2015.07.010. 26188590 10.1016/j.lfs.2015.07.010

[230] Foufelle F, Ferré P. [Unfolded protein response: its role in physiology and physiopathology]. Med Sci (Paris). 2007;23(3):291–6. La réponse UPR: son rôle physiologique et physiopathologique. 10.1051/medsci/2007233291. 10.1051/medsci/2007233291 17349291

[231] Zhang XQ, Xu CF, Yu CH, Chen WX, Li YM. Role of endoplasmic reticulum stress in the pathogenesis of nonalcoholic fatty liver disease. World J Gastroenterol. 2014;20(7):1768–76. 10.3748/wjg.v20.i7.1768. 24587654 10.3748/wjg.v20.i7.1768 PMC3930975

[232] Jegatheesan P, De Bandt JP. Fructose and NAFLD: The Multifaceted Aspects of Fructose Metabolism. Nutrients. 2017;9(3). 10.3390/nu9030230. 10.3390/nu9030230 PMC5372893 28273805

[233] Chen Q, Wang T, Li J, Wang S, Qiu F, Yu H, et al. Effects of Natural Products on Fructose-Induced Nonalcoholic Fatty Liver Disease (NAFLD). Nutrients. 2017;9(2). 10.3390/nu9020096. 10.3390/nu9020096 PMC5331527 28146130

[234] Alwahsh SM, Xu M, Schultze FC, Wilting J, Mihm S, Raddatz D, et al. Combination of alcohol and fructose exacerbates metabolic imbalance in terms of hepatic damage, dyslipidemia, and insulin resistance in rats. PLoS One. 2014;9(8):e104220. 10.1371/journal.pone.0104220. 25101998 10.1371/journal.pone.0104220 PMC4125190

[235] Novelle MG, Bravo SB, Deshons M, Iglesias C, García-Vence M, Annells R, et al. Impact of liver-specific GLUT8 silencing on fructose-induced inflammation and omega oxidation. iScience. 2021;24(2):102071. 10.1016/j.isci.2021.102071. 10.1016/j.isci.2021.102071 PMC7856473 33554072

[236] Joosten LAB, Crişan TO, Bjornstad P, Johnson RJ. Asymptomatic hyperuricaemia: a silent activator of the innate immune system. Nat Rev Rheumatol. 2020;16(2):75–86. 10.1038/s41584-019-0334-3. 31822862 10.1038/s41584-019-0334-3 PMC7075706

[237] Jhang JJ, Cheng YT, Ho CY, Yen GC. Monosodium urate crystals trigger Nrf2- and heme oxygenase-1-dependent inflammation in THP-1 cells. Cell Mol Immunol. 2015;12(4):424–34. 10.1038/cmi.2014.65. 25109682 10.1038/cmi.2014.65 PMC4496538

[238] Zhang X, Zhang JH, Chen XY, Hu QH, Wang MX, Jin R, et al. Reactive oxygen species-induced TXNIP drives fructose-mediated hepatic inflammation and lipid accumulation through NLRP3 inflammasome activation. Antioxid Redox Signal. 2015;22(10):848–70. 10.1089/ars.2014.5868. 25602171 10.1089/ars.2014.5868 PMC4367240

[239] Zhao XJ, Yu HW, Yang YZ, Wu WY, Chen TY, Jia KK, et al. Polydatin prevents fructose-induced liver inflammation and lipid deposition through increasing miR-200a to regulate Keap1/Nrf2 pathway. Redox Biol. 2018;18:124–37. 10.1016/j.redox.2018.07.002. 30014902 10.1016/j.redox.2018.07.002 PMC6068203

[240] Ramadori G, Armbrust T. Cytokines in the liver. Eur J Gastroenterol Hepatol. 2001;13(7):777–84. 10.1097/00042737-200107000-00004. 11474306 10.1097/00042737-200107000-00004

[241] Ju C, Tacke F. Hepatic macrophages in homeostasis and liver diseases: from pathogenesis to novel therapeutic strategies. Cell Mol Immunol. 2016;13(3):316–27. 10.1038/cmi.2015.104. 26908374 10.1038/cmi.2015.104 PMC4856798

[242] Traeger T, Mikulcak M, Eipel C, Abshagen K, Diedrich S, Heidecke CD, et al. Kupffer cell depletion reduces hepatic inflammation and apoptosis but decreases survival in abdominal sepsis. Eur J Gastroenterol Hepatol. 2010;22(9):1039–49. 10.1097/MEG.0b013e32833847db. 20300005 10.1097/MEG.0b013e32833847db

[243] Jensen VS, Hvid H, Damgaard J, Nygaard H, Ingvorsen C, Wulff EM, et al. Dietary fat stimulates development of NAFLD more potently than dietary fructose in Sprague-Dawley rats. Diabetol Metab Syndr. 2018;10:4. 10.1186/s13098-018-0307-8. 29410708 10.1186/s13098-018-0307-8 PMC5781341

[244] Muriel P, López-Sánchez P, Ramos-Tovar E. Fructose and the Liver. Int J Mol Sci. 2021;22(13). 10.3390/ijms22136969. 10.3390/ijms22136969 PMC8267750 34203484

[245] Baffy G. Kupffer cells in non-alcoholic fatty liver disease: the emerging view. J Hepatol. 2009;51(1):212–23. 10.1016/j.jhep.2009.03.008. 19447517 10.1016/j.jhep.2009.03.008 PMC2694233

[246] Rutkowsky JM, Knotts TA, Ono-Moore KD, McCoin CS, Huang S, Schneider D, et al. Acylcarnitines activate proinflammatory signaling pathways. Am J Physiol Endocrinol Metab. 2014;306(12):E1378-87. 10.1152/ajpendo.00656.2013. 24760988 10.1152/ajpendo.00656.2013 PMC4059985

[247] Softic S, Meyer JG, Wang GX, Gupta MK, Batista TM, Lauritzen H, et al. Dietary Sugars Alter Hepatic Fatty Acid Oxidation via Transcriptional and Post-translational Modifications of Mitochondrial Proteins. Cell Metab. 2019;30(4):735-53.e4. 10.1016/j.cmet.2019.09.003. 31577934 10.1016/j.cmet.2019.09.003 PMC7816129

[248] Li N, Zhao H. Role of Carnitine in Non-alcoholic Fatty Liver Disease and Other Related Diseases: An Update. Front Med (Lausanne). 2021;8:689042. 10.3389/fmed.2021.689042. 34434943 10.3389/fmed.2021.689042 PMC8381051

[249] Mazzoli A, Spagnuolo MS, Gatto C, Nazzaro M, Cancelliere R, Crescenzo R, et al. Adipose Tissue and Brain Metabolic Responses to Western Diet-Is There a Similarity between the Two? Int J Mol Sci. 2020;21(3) 10.3390/ijms21030786. 10.3390/ijms21030786 PMC7036881 31991770

[250] Spagnuolo MS, Pallottini V, Mazzoli A, Iannotta L, Tonini C, Morone B, et al. A Short-Term Western Diet Impairs Cholesterol Homeostasis and Key Players of Beta Amyloid Metabolism in Brain of Middle Aged Rats. Mol Nutr Food Res. 2020;64(16):e2000541. 10.1002/mnfr.202000541. 32579784 10.1002/mnfr.202000541

[251] Ting KKY. Fructose overconsumption-induced reprogramming of microglia metabolism and function. Front Immunol. 2024;15:1375453. 10.3389/fimmu.2024.1375453. 38596671 10.3389/fimmu.2024.1375453 PMC11002174

[252] Hassel B, Elsais A, Frøland AS, Taubøll E, Gjerstad L, Quan Y, et al. Uptake and metabolism of fructose by rat neocortical cells in vivo and by isolated nerve terminals in vitro. J Neurochem. 2015;133(4):572–81. 10.1111/jnc.13079. 25708447 10.1111/jnc.13079 PMC4427911

[253] Shu HJ, Isenberg K, Cormier RJ, Benz A, Zorumski CF. Expression of fructose sensitive glucose transporter in the brains of fructose-fed rats. Neuroscience. 2006;140(3):889–95. 10.1016/j.neuroscience.2006.02.071. 16581195 10.1016/j.neuroscience.2006.02.071

[254] Hsu TM, Kanoski SE. Blood-brain barrier disruption: mechanistic links between Western diet consumption and dementia. Front Aging Neurosci. 2014;6:88. 10.3389/fnagi.2014.00088. 24847262 10.3389/fnagi.2014.00088 PMC4023063

[255] Spagnuolo MS, Iossa S, Cigliano L. Sweet but Bitter: Focus on Fructose Impact on Brain Function in Rodent Models. Nutrients. 2020;13(1). 10.3390/nu13010001. 10.3390/nu13010001 PMC7821920 33374894

[256] Jiménez-Maldonado A, Ying Z, Byun HR, Gomez-Pinilla F. Short-term fructose ingestion affects the brain independently from establishment of metabolic syndrome. Biochim Biophys Acta Mol Basis Dis. 2018;1864(1):24–33. 10.1016/j.bbadis.2017.10.012. 29017895 10.1016/j.bbadis.2017.10.012 PMC5705281

[257] Leuner B, Gould E. Structural plasticity and hippocampal function. Annu Rev Psychol. 2010;61(111–40):c1-3. 10.1146/annurev.psych.093008.100359. 10.1146/annurev.psych.093008.100359 PMC3012424 19575621

[258] Stranahan AM, Norman ED, Lee K, Cutler RG, Telljohann RS, Egan JM, et al. Diet-induced insulin resistance impairs hippocampal synaptic plasticity and cognition in middle-aged rats. Hippocampus. 2008;18(11):1085–8. 10.1002/hipo.20470. 18651634 10.1002/hipo.20470 PMC2694409

[259] Lakhan SE, Kirchgessner A. The emerging role of dietary fructose in obesity and cognitive decline. Nutr J. 2013;12:114. 10.1186/1475-2891-12-114. 23924506 10.1186/1475-2891-12-114 PMC3751294

[260] Agrawal R, Gomez-Pinilla F. “Metabolic syndrome” in the brain: deficiency in omega-3 fatty acid exacerbates dysfunctions in insulin receptor signalling and cognition. J Physiol. 2012;590(10):2485–99. 10.1113/jphysiol.2012.230078. 22473784 10.1113/jphysiol.2012.230078 PMC3424766

[261] Mazzoli A, Spagnuolo MS, Nazzaro M, Gatto C, Iossa S, Cigliano L. Fructose Removal from the Diet Reverses Inflammation, Mitochondrial Dysfunction, and Oxidative Stress in Hippocampus. Antioxidants (Basel). 2021;10(3). 10.3390/antiox10030487. 10.3390/antiox10030487 PMC8003595 33804637

[262] Shao X, Lu W, Gao F, Li D, Hu J, Li Y, et al. Uric Acid Induces Cognitive Dysfunction through Hippocampal Inflammation in Rodents and Humans. J Neurosci. 2016;36(43):10990–1005. 10.1523/jneurosci.1480-16.2016. 27798180 10.1523/JNEUROSCI.1480-16.2016 PMC6705652

[263] Rai AK, Jaiswal N, Maurya CK, Sharma A, Ahmad I, Ahmad S, et al. Fructose-induced AGEs-RAGE signaling in skeletal muscle contributes to impairment of glucose homeostasis. J Nutr Biochem. 2019;71:35–44. 10.1016/j.jnutbio.2019.05.016. 31272030 10.1016/j.jnutbio.2019.05.016

[264] Aragno M, Mastrocola R, Medana C, Restivo F, Catalano MG, Pons N, et al. Up-regulation of advanced glycated products receptors in the brain of diabetic rats is prevented by antioxidant treatment. Endocrinology. 2005;146(12):5561–7. 10.1210/en.2005-0712. 16166220 10.1210/en.2005-0712

[265] Allaman I, Bélanger M, Magistretti PJ. Methylglyoxal, the dark side of glycolysis. Front Neurosci. 2015;9:23. 10.3389/fnins.2015.00023. 25709564 10.3389/fnins.2015.00023 PMC4321437

[266] Mastrocola R, Nigro D, Cento AS, Chiazza F, Collino M, Aragno M. High-fructose intake as risk factor for neurodegeneration: Key role for carboxy methyllysine accumulation in mice hippocampal neurons. Neurobiol Dis. 2016;89:65–75. 10.1016/j.nbd.2016.02.005. 26851500 10.1016/j.nbd.2016.02.005

[267] Hanisch UK, Kettenmann H. Microglia: active sensor and versatile effector cells in the normal and pathologic brain. Nat Neurosci. 2007;10(11):1387–94. 10.1038/nn1997. 17965659 10.1038/nn1997

[268] Konsman JP, Kelley K, Dantzer R. Temporal and spatial relationships between lipopolysaccharide-induced expression of Fos, interleukin-1beta and inducible nitric oxide synthase in rat brain. Neuroscience. 1999;89(2):535–48. 10.1016/s0306-4522(98)00368-6. 10077334 10.1016/s0306-4522(98)00368-6

[269] Marty V, El Hachmane M, Amédée T. Dual modulation of synaptic transmission in the nucleus tractus solitarius by prostaglandin E2 synthesized downstream of IL-1beta. Eur J Neurosci. 2008;27(12):3132–50. 10.1111/j.1460-9568.2008.06296.x. 18598258 10.1111/j.1460-9568.2008.06296.x

[270] Cigliano L, Spagnuolo MS, Crescenzo R, Cancelliere R, Iannotta L, Mazzoli A, et al. Short-Term Fructose Feeding Induces Inflammation and Oxidative Stress in the Hippocampus of Young and Adult Rats. Mol Neurobiol. 2018;55(4):2869–83. 10.1007/s12035-017-0518-2. 28455700 10.1007/s12035-017-0518-2

[271] Yamazaki M, Yamada H, Munetsuna E, Ishikawa H, Mizuno G, Mukuda T, et al. Excess maternal fructose consumption impairs hippocampal function in offspring via epigenetic modification of BDNF promoter. Faseb j. 2018;32(5):2549–62. 10.1096/fj.201700783RR. 29401579 10.1096/fj.201700783RR

[272] Haydon PG, Carmignoto G. Astrocyte control of synaptic transmission and neurovascular coupling. Physiol Rev. 2006;86(3):1009–31. 10.1152/physrev.00049.2005. 16816144 10.1152/physrev.00049.2005

[273] Sofroniew MV, Vinters HV. Astrocytes: biology and pathology. Acta Neuropathol. 2010;119(1):7–35. 10.1007/s00401-009-0619-8. 20012068 10.1007/s00401-009-0619-8 PMC2799634

[274] López M, Varela L, Vázquez MJ, Rodríguez-Cuenca S, González CR, Velagapudi VR, et al. Hypothalamic AMPK and fatty acid metabolism mediate thyroid regulation of energy balance. Nat Med. 2010;16(9):1001–8. 10.1038/nm.2207. 20802499 10.1038/nm.2207 PMC2935934

[275] Mayer CM, Fick LJ, Gingerich S, Belsham DD. Hypothalamic cell lines to investigate neuroendocrine control mechanisms. Front Neuroendocrinol. 2009;30(3):405–23. 10.1016/j.yfrne.2009.03.005. 19341762 10.1016/j.yfrne.2009.03.005

[276] Li JM, Ge CX, Xu MX, Wang W, Yu R, Fan CY, et al. Betaine recovers hypothalamic neural injury by inhibiting astrogliosis and inflammation in fructose-fed rats. Mol Nutr Food Res. 2015;59(2):189–202. 10.1002/mnfr.201400307. 25303559 10.1002/mnfr.201400307

[277] Giedd JN, Blumenthal J, Jeffries NO, Castellanos FX, Liu H, Zijdenbos A, et al. Brain development during childhood and adolescence: a longitudinal MRI study. Nat Neurosci. 1999;2(10):861–3. 10.1038/13158. 10491603 10.1038/13158

[278] Manitt C, Eng C, Pokinko M, Ryan RT, Torres-Berrío A, Lopez JP, et al. dcc orchestrates the development of the prefrontal cortex during adolescence and is altered in psychiatric patients. Transl Psychiatry. 2013;3(12):e338. 10.1038/tp.2013.105. 24346136 10.1038/tp.2013.105 PMC4030324

[279] Morin JP, Rodríguez-Durán LF, Guzmán-Ramos K, Perez-Cruz C, Ferreira G, Diaz-Cintra S, et al. Palatable Hyper-Caloric Foods Impact on Neuronal Plasticity. Front Behav Neurosci. 2017;11:19. 10.3389/fnbeh.2017.00019. 28261067 10.3389/fnbeh.2017.00019 PMC5306218

[280] Reichelt AC. Adolescent Maturational Transitions in the Prefrontal Cortex and Dopamine Signaling as a Risk Factor for the Development of Obesity and High Fat/High Sugar Diet Induced Cognitive Deficits. Front Behav Neurosci. 2016;10:189. 10.3389/fnbeh.2016.00189. 27790098 10.3389/fnbeh.2016.00189 PMC5061823

[281] Spagnuolo MS, Bergamo P, Crescenzo R, Iannotta L, Treppiccione L, Iossa S, et al. Brain Nrf2 pathway, autophagy, and synaptic function proteins are modulated by a short-term fructose feeding in young and adult rats. Nutr Neurosci. 2020;23(4):309–20. 10.1080/1028415x.2018.1501532. 30039750 10.1080/1028415X.2018.1501532

[282] Oudot C, Lajoix AD, Jover B, Rugale C. Dietary sodium restriction prevents kidney damage in high fructose-fed rats. Kidney Int. 2013;83(4):674–83. 10.1038/ki.2012.478. 23344470 10.1038/ki.2012.478

[283] Singh AK, Amlal H, Haas PJ, Dringenberg U, Fussell S, Barone SL, et al. Fructose-induced hypertension: essential role of chloride and fructose absorbing transporters PAT1 and Glut5. Kidney Int. 2008;74(4):438–47. 10.1038/ki.2008.184. 18496516 10.1038/ki.2008.184 PMC10947803

[284] Nakayama T, Kosugi T, Gersch M, Connor T, Sanchez-Lozada LG, Lanaspa MA, et al. Dietary fructose causes tubulointerstitial injury in the normal rat kidney. Am J Physiol Renal Physiol. 2010;298(3):F712-20. 10.1152/ajprenal.00433.2009. 20071464 10.1152/ajprenal.00433.2009 PMC2838595

[285] Wang W, Ding XQ, Gu TT, Song L, Li JM, Xue QC, et al. Pterostilbene and allopurinol reduce fructose-induced podocyte oxidative stress and inflammation via microRNA-377. Free Radic Biol Med. 2015;83:214–26. 10.1016/j.freeradbiomed.2015.02.029. 25746774 10.1016/j.freeradbiomed.2015.02.029

[286] Skeel A, Yoshimura T, Showalter SD, Tanaka S, Appella E, Leonard EJ. Macrophage stimulating protein: purification, partial amino acid sequence, and cellular activity. J Exp Med. 1991;173(5):1227–34. 10.1084/jem.173.5.1227. 1827141 10.1084/jem.173.5.1227 PMC2118857

[287] Glushakova O, Kosugi T, Roncal C, Mu W, Heinig M, Cirillo P, et al. Fructose induces the inflammatory molecule ICAM-1 in endothelial cells. J Am Soc Nephrol. 2008;19(9):1712–20. 10.1681/asn.2007121304. 18508964 10.1681/ASN.2007121304 PMC2518440

[288] De Fea K, Roth RA. Protein kinase C modulation of insulin receptor substrate-1 tyrosine phosphorylation requires serine 612. Biochemistry. 1997;36(42):12939–47. 10.1021/bi971157f. 9335553 10.1021/bi971157f

[289] Hotamisligil GS, Shargill NS, Spiegelman BM. Adipose expression of tumor necrosis factor-alpha: direct role in obesity-linked insulin resistance. Science. 1993;259(5091):87–91. 10.1126/science.7678183. 7678183 10.1126/science.7678183

[290] Fantuzzi G, Faggioni R. Leptin in the regulation of immunity, inflammation, and hematopoiesis. J Leukoc Biol. 2000;68(4):437–46. 11037963

[291] DiNicolantonio JJ, Mehta V, Onkaramurthy N, O’Keefe JH. Fructose-induced inflammation and increased cortisol: A new mechanism for how sugar induces visceral adiposity. Prog Cardiovasc Dis. 2018;61(1):3–9. 10.1016/j.pcad.2017.12.001. 29225114 10.1016/j.pcad.2017.12.001

[292] Pokrywczynska M, Flisinski M, Jundzill A, Krzyzanowska S, Brymora A, Deptula A, et al. Impact of fructose diet and renal failure on the function of pancreatic islets. Pancreas. 2014;43(5):801–8. 10.1097/mpa.0000000000000111. 24739489 10.1097/MPA.0000000000000111

[293] Tian YF, He CT, Chen YT, Hsieh PS. Lipoic acid suppresses portal endotoxemia-induced steatohepatitis and pancreatic inflammation in rats. World J Gastroenterol. 2013;19(18):2761–71. 10.3748/wjg.v19.i18.2761. 23687413 10.3748/wjg.v19.i18.2761 PMC3653150

[294] Tappy L. Fructose-containing caloric sweeteners as a cause of obesity and metabolic disorders. J Exp Biol. 2018;221(Pt Suppl 1). 10.1242/jeb.164202. 10.1242/jeb.164202 29514881

[295] Bray GA. Soft drink consumption and obesity: it is all about fructose. Curr Opin Lipidol. 2010;21(1):51–7. 10.1097/MOL.0b013e3283346ca2. 19956074 10.1097/MOL.0b013e3283346ca2

[296] Cui Y, Tian J, Wang Z, Guo H, Zhang H, Wang Z, et al. Fructose-Induced mTORC1 Activation Promotes Pancreatic Cancer Progression through Inhibition of Autophagy. Cancer Res. 2023;83(24):4063–79. 10.1158/0008-5472.Can-23-0464. 37738413 10.1158/0008-5472.CAN-23-0464 PMC10722142

[297] Peng C, Yang P, Zhang D, Jin C, Peng W, Wang T, et al. KHK-A promotes fructose-dependent colorectal cancer liver metastasis by facilitating the phosphorylation and translocation of PKM2. Acta Pharm Sin B. 2024;14(7):2959–76. 10.1016/j.apsb.2024.04.024. 39027256 10.1016/j.apsb.2024.04.024 PMC11252482

[298] Fang JH, Chen JY, Zheng JL, Zeng HX, Chen JG, Wu CH, et al. Fructose Metabolism in Tumor Endothelial Cells Promotes Angiogenesis by Activating AMPK Signaling and Mitochondrial Respiration. Cancer Res. 2023;83(8):1249–63. 10.1158/0008-5472.Can-22-1844. 36715635 10.1158/0008-5472.CAN-22-1844

[299] Semnani-Azad Z, Khan TA, Blanco Mejia S, de Souza RJ, Leiter LA, Kendall CWC, et al. Association of Major Food Sources of Fructose-Containing Sugars With Incident Metabolic Syndrome: A Systematic Review and Meta-analysis. JAMA Netw Open. 2020;3(7):e209993. 10.1001/jamanetworkopen.2020.9993. 32644139 10.1001/jamanetworkopen.2020.9993 PMC7348689

[300] Aoun R, Chokor FAZ, Taktouk M, Nasrallah M, Ismaeel H, Tamim H, et al. Dietary fructose and its association with the metabolic syndrome in Lebanese healthy adults: a cross-sectional study. Diabetol Metab Syndr. 2022;14(1):29. 10.1186/s13098-022-00800-5. 35139893 10.1186/s13098-022-00800-5 PMC8827166

[301] Pang S, Song P, Sun X, Qi W, Yang C, Song G, et al. Dietary fructose and risk of metabolic syndrome in Chinese residents aged 45 and above: results from the China National Nutrition and Health Survey. Nutr J. 2021;20(1):83. 10.1186/s12937-021-00739-9. 34602079 10.1186/s12937-021-00739-9 PMC8489071

[302] Aune D, Chan DSM, Vieira AR, Navarro Rosenblatt DA, Vieira R, Greenwood DC, et al. Dietary fructose, carbohydrates, glycemic indices and pancreatic cancer risk: a systematic review and meta-analysis of cohort studies. Ann Oncol. 2012;23(10):2536–46. 10.1093/annonc/mds076. 22539563 10.1093/annonc/mds076

[303] Devall MA, Eaton S, Hu G, Sun X, Jakum E, Venkatesh S, et al. Association between dietary fructose and human colon DNA methylation: implication for racial disparities in colorectal cancer risk using a cross-sectional study. Am J Clin Nutr. 2025;121(3):522–34. 10.1016/j.ajcnut.2025.01.005. 39788295 10.1016/j.ajcnut.2025.01.005 PMC11923427

[304] Arthur RS, Kirsh VA, Mossavar-Rahmani Y, Xue X, Rohan TE. Sugar-containing beverages and their association with risk of breast, endometrial, ovarian and colorectal cancers among Canadian women. Cancer Epidemiol. 2021;70:101855. 10.1016/j.canep.2020.101855. 33220638 10.1016/j.canep.2020.101855

[305] Farvid MS, Barnett JB, Spence ND, Rosner BA, Holmes MD. Types of carbohydrate intake and breast cancer survival. Eur J Nutr. 2021;60(8):4565–77. 10.1007/s00394-021-02517-z. 34152461 10.1007/s00394-021-02517-z PMC9938676

[306] Makarem N, Bandera EV, Lin Y, Jacques PF, Hayes RB, Parekh N. Consumption of Sugars, Sugary Foods, and Sugary Beverages in Relation to Adiposity-Related Cancer Risk in the Framingham Offspring Cohort (1991–2013). Cancer Prev Res (Phila). 2018;11(6):347–58. 10.1158/1940-6207.Capr-17-0218. 29674390 10.1158/1940-6207.CAPR-17-0218 PMC7225083

[307] Huang C, Liang Z, Ma J, Hu D, Yao F, Qin P. Total sugar, added sugar, fructose, and sucrose intake and all-cause, cardiovascular, and cancer mortality: A systematic review and dose-response meta-analysis of prospective cohort studies. Nutrition. 2023;111:112032. 10.1016/j.nut.2023.112032. 37182401 10.1016/j.nut.2023.112032

[308] Carreño D, Corro N, Torres-Estay V, Véliz LP, Jaimovich R, Cisternas P, et al. Fructose and prostate cancer: toward an integrated view of cancer cell metabolism. Prostate Cancer Prostatic Dis. 2019;22(1):49–58. 10.1038/s41391-018-0072-7. 30104655 10.1038/s41391-018-0072-7

[309] Strober JW, Brady MJ. Dietary Fructose Consumption and Triple-Negative Breast Cancer Incidence. Front Endocrinol (Lausanne). 2019;10:367. 10.3389/fendo.2019.00367. 31244777 10.3389/fendo.2019.00367 PMC6581676

[310] Jiang H, Lin Q, Ma L, Luo S, Jiang X, Fang J, et al. Fructose and fructose kinase in cancer and other pathologies. J Genet Genomics. 2021;48(7):531–9. 10.1016/j.jgg.2021.06.006. 34326012 10.1016/j.jgg.2021.06.006

[311] Liu H, Heaney AP. Refined fructose and cancer. Expert Opin Ther Targets. 2011;15(9):1049–59. 10.1517/14728222.2011.588208. 21623683 10.1517/14728222.2011.588208

[312] Boroughs LK, DeBerardinis RJ. Metabolic pathways promoting cancer cell survival and growth. Nat Cell Biol. 2015;17(4):351–9. 10.1038/ncb3124. 25774832 10.1038/ncb3124 PMC4939711

[313] Kuehm LM, Khojandi N, Piening A, Klevorn LE, Geraud SC, McLaughlin NR, et al. Fructose Promotes Cytoprotection in Melanoma Tumors and Resistance to Immunotherapy. Cancer Immunol Res. 2021;9(2):227–38. 10.1158/2326-6066.Cir-20-0396. 33023966 10.1158/2326-6066.CIR-20-0396 PMC7864871

[314] Fowle-Grider R, Rowles JL 3rd, Shen I, Wang Y, Schwaiger-Haber M, Dunham AJ, et al. Dietary fructose enhances tumour growth indirectly via interorgan lipid transfer. Nature. 2024;636(8043):737–44. 10.1038/s41586-024-08258-3. 39633044 10.1038/s41586-024-08258-3 PMC12276860

[315] Yan H, Wang Z, Teng D, Chen X, Zhu Z, Chen H, et al. Hexokinase 2 senses fructose in tumor-associated macrophages to promote colorectal cancer growth. Cell Metab. 2024;36(11):2449-67.e6. 10.1016/j.cmet.2024.10.002. 39471815 10.1016/j.cmet.2024.10.002

[316] Chen WL, Wang YY, Zhao A, Xia L, Xie G, Su M, et al. Enhanced Fructose Utilization Mediated by SLC2A5 Is a Unique Metabolic Feature of Acute Myeloid Leukemia with Therapeutic Potential. Cancer Cell. 2016;30(5):779–91. 10.1016/j.ccell.2016.09.006. 27746145 10.1016/j.ccell.2016.09.006 PMC5496656

[317] Kang YL, Kim J, Kwak SB, Kim YS, Huh J, Park JW. The polyol pathway and nuclear ketohexokinase A signaling drive hyperglycemia-induced metastasis of gastric cancer. Exp Mol Med. 2024;56(1):220–34. 10.1038/s12276-023-01153-3. 38200154 10.1038/s12276-023-01153-3 PMC10834943

[318] Zhang Y, Yu X, Bao R, Huang H, Gu C, Lv Q, et al. Cell Metab. 2023;35(12):2107-18.e6. 10.1016/j.cmet.2023.09.011. 37863051 10.1016/j.cmet.2023.09.011

[319] Dewdney B, Alanazy M, Gillman R, Walker S, Wankell M, Qiao L, et al. The effects of fructose and metabolic inhibition on hepatocellular carcinoma. Sci Rep. 2020;10(1):16769. 10.1038/s41598-020-73653-5. 33028928 10.1038/s41598-020-73653-5 PMC7541473

[320] Joh HK, Lee DH, Hur J, Nimptsch K, Chang Y, Joung H, et al. Simple Sugar and Sugar-Sweetened Beverage Intake During Adolescence and Risk of Colorectal Cancer Precursors. Gastroenterology. 2021;161(1):128-42.e20. 10.1053/j.gastro.2021.03.028. 33753105 10.1053/j.gastro.2021.03.028 PMC8238879

[321] Tasevska N, Jiao L, Cross AJ, Kipnis V, Subar AF, Hollenbeck A, et al. Sugars in diet and risk of cancer in the NIH-AARP Diet and Health Study. Int J Cancer. 2012;130(1):159–69. 10.1002/ijc.25990. 21328345 10.1002/ijc.25990 PMC3494407

[322] Meyerhardt JA, Sato K, Niedzwiecki D, Ye C, Saltz LB, Mayer RJ, et al. Dietary glycemic load and cancer recurrence and survival in patients with stage III colon cancer: findings from CALGB 89803. J Natl Cancer Inst. 2012;104(22):1702–11. 10.1093/jnci/djs399. 23136358 10.1093/jnci/djs399 PMC3502194

[323] Su C, Li H, Gao W. GLUT5 increases fructose utilization and promotes tumor progression in glioma. Biochem Biophys Res Commun. 2018;500(2):462–9. 10.1016/j.bbrc.2018.04.103. 29660339 10.1016/j.bbrc.2018.04.103

[324] Carreño DV, Corro NB, Cerda-Infante JF, Echeverría CE, Asencio-Barría CA, Torres-Estay VA, et al. Dietary Fructose Promotes Prostate Cancer Growth. Cancer Res. 2021;81(11):2824–32. 10.1158/0008-5472.Can-19-0456. 33762358 10.1158/0008-5472.CAN-19-0456

[325] Chen WL, Jin X, Wang M, Liu D, Luo Q, Tian H, et al. GLUT5-mediated fructose utilization drives lung cancer growth by stimulating fatty acid synthesis and AMPK/mTORC1 signaling. JCI Insight. 2020;5(3). 10.1172/jci.insight.131596. 10.1172/jci.insight.131596 PMC7098789 32051337

[326] Gao W, Li N, Li Z, Xu J, Su C. Ketohexokinase is involved in fructose utilization and promotes tumor progression in glioma. Biochem Biophys Res Commun. 2018;503(3):1298–306. 10.1016/j.bbrc.2018.07.040. 30031605 10.1016/j.bbrc.2018.07.040

[327] Tao QF, Yuan SX, Yang F, Yang S, Yang Y, Yuan JH, et al. Aldolase B inhibits metastasis through Ten-Eleven Translocation 1 and serves as a prognostic biomarker in hepatocellular carcinoma. Mol Cancer. 2015;14:170. 10.1186/s12943-015-0437-7. 26376879 10.1186/s12943-015-0437-7 PMC4574028

[328] Ducker GS, Rabinowitz JD. One-Carbon Metabolism in Health and Disease. Cell Metab. 2017;25(1):27–42. 10.1016/j.cmet.2016.08.009. 27641100 10.1016/j.cmet.2016.08.009 PMC5353360

[329] Pacold ME, Brimacombe KR, Chan SH, Rohde JM, Lewis CA, Swier LJ, et al. A PHGDH inhibitor reveals coordination of serine synthesis and one-carbon unit fate. Nat Chem Biol. 2016;12(6):452–8. 10.1038/nchembio.2070. 27110680 10.1038/nchembio.2070 PMC4871733

[330] Sullivan MR, Mattaini KR, Dennstedt EA, Nguyen AA, Sivanand S, Reilly MF, et al. Increased Serine Synthesis Provides an Advantage for Tumors Arising in Tissues Where Serine Levels Are Limiting. Cell Metab. 2019;29(6):1410-21.e4. 10.1016/j.cmet.2019.02.015. 30905671 10.1016/j.cmet.2019.02.015 PMC6551255

[331] Possemato R, Marks KM, Shaul YD, Pacold ME, Kim D, Birsoy K, et al. Functional genomics reveal that the serine synthesis pathway is essential in breast cancer. Nature. 2011;476(7360):346–50. 10.1038/nature10350. 21760589 10.1038/nature10350 PMC3353325

[332] Wei L, Lee D, Law CT, Zhang MS, Shen J, Chin DW, et al. Genome-wide CRISPR/Cas9 library screening identified PHGDH as a critical driver for Sorafenib resistance in HCC. Nat Commun. 2019;10(1):4681. 10.1038/s41467-019-12606-7. 31615983 10.1038/s41467-019-12606-7 PMC6794322

[333] Sano H, Nakamura A, Yamane M, Niwa H, Nishimura T, Araki K, et al. The polyol pathway is an evolutionarily conserved system for sensing glucose uptake. PLoS Biol. 2022;20(6):e3001678. 10.1371/journal.pbio.3001678. 35687590 10.1371/journal.pbio.3001678 PMC9223304

[334] Schwab A, Siddiqui A, Vazakidou ME, Napoli F, Böttcher M, Menchicchi B, et al. Polyol Pathway Links Glucose Metabolism to the Aggressiveness of Cancer Cells. Cancer Res. 2018;78(7):1604–18. 10.1158/0008-5472.Can-17-2834. 29343522 10.1158/0008-5472.CAN-17-2834

[335] Douard V, Ferraris RP. The role of fructose transporters in diseases linked to excessive fructose intake. J Physiol. 2013;591(2):401–14. 10.1113/jphysiol.2011.215731. 23129794 10.1113/jphysiol.2011.215731 PMC3577529

[336] Liu H, Huang D, McArthur DL, Boros LG, Nissen N, Heaney AP. Fructose induces transketolase flux to promote pancreatic cancer growth. Cancer Res. 2010;70(15):6368–76. 10.1158/0008-5472.Can-09-4615. 20647326 10.1158/0008-5472.CAN-09-4615

[337] Lanaspa MA, Ishimoto T, Cicerchi C, Tamura Y, Roncal-Jimenez CA, Chen W, et al. Endogenous fructose production and fructokinase activation mediate renal injury in diabetic nephropathy. J Am Soc Nephrol. 2014;25(11):2526–38. 10.1681/asn.2013080901. 24876114 10.1681/ASN.2013080901 PMC4214522

[338] Port AM, Ruth MR, Istfan NW. Fructose consumption and cancer: is there a connection? Curr Opin Endocrinol Diabetes Obes. 2012;19(5):367–74. 10.1097/MED.0b013e328357f0cb. 22922366 10.1097/MED.0b013e328357f0cb

[339] Song A, Mao Y, Wei H. GLUT5: structure, functions, diseases and potential applications. Acta Biochim Biophys Sin (Shanghai). 2023;55(10):1519–38. 10.3724/abbs.2023158. 37674366 10.3724/abbs.2023158 PMC10582729

[340] Hadzi-Petrushev N, Stojchevski R, Jakimovska A, Stamenkovska M, Josifovska S, Stamatoski A, et al. GLUT5-overexpression-related tumorigenic implications. Mol Med. 2024;30(1):114. 10.1186/s10020-024-00879-8. 39107723 10.1186/s10020-024-00879-8 PMC11304774

[341] George Thompson AM, Ursu O, Babkin P, Iancu CV, Whang A, Oprea TI, et al. Discovery of a specific inhibitor of human GLUT5 by virtual screening and in vitro transport evaluation. Sci Rep. 2016;6:24240. 10.1038/srep24240. 27074918 10.1038/srep24240 PMC4831007

[342] Park SH, Fadhul T, Conroy LR, Clarke HA, Sun RC, Wallenius K, et al. Knockdown of ketohexokinase versus inhibition of its kinase activity exert divergent effects on fructose metabolism. JCI Insight. 2024;9(23). 10.1172/jci.insight.184396. 10.1172/jci.insight.184396 PMC11623947 39418102

[343] Huard K, Ahn K, Amor P, Beebe DA, Borzilleri KA, Chrunyk BA, et al. Discovery of Fragment-Derived Small Molecules for in Vivo Inhibition of Ketohexokinase (KHK). J Med Chem. 2017;60(18):7835–49. 10.1021/acs.jmedchem.7b00947. 28853885 10.1021/acs.jmedchem.7b00947

[344] Gutierrez JA, Liu W, Perez S, Xing G, Sonnenberg G, Kou K, et al. Pharmacologic inhibition of ketohexokinase prevents fructose-induced metabolic dysfunction. Mol Metab. 2021;48:101196. 10.1016/j.molmet.2021.101196. 33667726 10.1016/j.molmet.2021.101196 PMC8050029

[345] Futatsugi K, Smith AC, Tu M, Raymer B, Ahn K, Coffey SB, et al. Discovery of PF-06835919: A Potent Inhibitor of Ketohexokinase (KHK) for the Treatment of Metabolic Disorders Driven by the Overconsumption of Fructose. J Med Chem. 2020;63(22):13546–60. 10.1021/acs.jmedchem.0c00944. 32910646 10.1021/acs.jmedchem.0c00944

[346] Kazierad DJ, Chidsey K, Somayaji VR, Bergman AJ, Birnbaum MJ, Calle RA. Inhibition of ketohexokinase in adults with NAFLD reduces liver fat and inflammatory markers: A randomized phase 2 trial. Med. 2021;2(7):800-13.e3. 10.1016/j.medj.2021.04.007. 35590219 10.1016/j.medj.2021.04.007

[347] Zhu G, Li J, Lin X, Zhang Z, Hu T, Huo S, et al. Discovery of a Novel Ketohexokinase Inhibitor with Improved Drug Distribution in Target Tissue for the Treatment of Fructose Metabolic Disease. J Med Chem. 2023;66(19):13501–15. 10.1021/acs.jmedchem.3c00715. 37766386 10.1021/acs.jmedchem.3c00715

[348] Shen Z, Li Z, Liu Y, Li Y, Feng X, Zhan Y, et al. GLUT5-KHK axis-mediated fructose metabolism drives proliferation and chemotherapy resistance of colorectal cancer. Cancer Lett. 2022;534:215617. 10.1016/j.canlet.2022.215617. 35257833 10.1016/j.canlet.2022.215617

[349] Westerbeke FHM, Rios-Morales M, Attaye I, Nieuwdorp M. Fructose catabolism and its metabolic effects: Exploring host-microbiota interactions and the impact of ethnicity. J Physiol. 2025; 10.1113/jp287316. 10.1113/JP287316 PMC12704044 39805044

[350] Chen Q, Luo Y, Shen Y, Li X, Yang H, Li J, et al. Fructose corn syrup induces inflammatory injury and obesity by altering gut microbiota and gut microbiota-related arachidonic acid metabolism. J Nutr Biochem. 2024;124:109527. 10.1016/j.jnutbio.2023.109527. 37979711 10.1016/j.jnutbio.2023.109527

[351] Song G, Gan Q, Qi W, Wang Y, Xu M, Li Y. Fructose Stimulated Colonic Arginine and Proline Metabolism Dysbiosis, Altered Microbiota and Aggravated Intestinal Barrier Dysfunction in DSS-Induced Colitis Rats. Nutrients. 2023;15(3). 10.3390/nu15030782. 10.3390/nu15030782 PMC9921751 36771488

[352] Dong W, Huang Y, Shu Y, Fan X, Tian X, Yan Y, et al. Water extract of goji berries improves neuroinflammation induced by a high-fat and high-fructose diet based on the bile acid-mediated gut-brain axis pathway. Food Funct. 2023;14(18):8631–45. 10.1039/d3fo02651e. 37670564 10.1039/d3fo02651e

[353] Peng Y, Dong W, Chen G, Mi J, Lu L, Xie Z, et al. Anthocyanins from Lycium ruthenicum Murray Ameliorated High-Fructose Diet-Induced Neuroinflammation through the Promotion of the Integrity of the Intestinal Barrier and the Proliferation of Lactobacillus. J Agric Food Chem. 2023;71(6):2864–82. 10.1021/acs.jafc.2c06713. 36725206 10.1021/acs.jafc.2c06713

[354] Grant AM, Christie MR, Ashcroft SJ. Insulin release from human pancreatic islets in vitro. Diabetologia. 1980;19(2):114–7. 10.1007/bf00421856. 6998814 10.1007/BF00421856

[355] Hwang IS, Huang WC, Wu JN, Shian LR, Reaven GM. Effect of fructose-induced hypertension on the renin-angiotensin-aldosterone system and atrial natriuretic factor. Am J Hypertens. 1989;2(6 Pt 1):424–7. 10.1093/ajh/2.6.424. 2527043 10.1093/ajh/2.6.424

[356] Sun SZ, Flickinger BD, Williamson-Hughes PS, Empie MW. Lack of association between dietary fructose and hyperuricemia risk in adults. Nutr Metab (Lond). 2010;7:16. 10.1186/1743-7075-7-16. 20193069 10.1186/1743-7075-7-16 PMC2842271

[357] Ayoub-Charette S, Liu Q, Khan TA, Au-Yeung F, Blanco Mejia S, de Souza RJ, et al. Important food sources of fructose-containing sugars and incident gout: a systematic review and meta-analysis of prospective cohort studies. BMJ Open. 2019;9(5):e024171. 10.1136/bmjopen-2018-024171. 31061018 10.1136/bmjopen-2018-024171 PMC6502023

[358] Saxena AR, Lyle SA, Khavandi K, Qiu R, Whitlock M, Esler WP, et al. A phase 2a, randomized, double-blind, placebo-controlled, three-arm, parallel-group study to assess the efficacy, safety, tolerability and pharmacodynamics of PF-06835919 in patients with non-alcoholic fatty liver disease and type 2 diabetes. Diabetes Obes Metab. 2023;25(4):992–1001. 10.1111/dom.14946. 36515213 10.1111/dom.14946

